# Supplementary material for: Health system interventions to integrate genetic testing in routine oncology services: A systematic review
Source: PLoS One. 2021 May 19;16(5):e0250379. doi: 10.1371/journal.pone.0250379 (PMC8133413; doi:10.1371/journal.pone.0250379)
Supplement: S1 Table — (PDF) [file pone.0250379.s001.pdf]

S1 Table. Search Strategy Medline, Embase, PsychINFO (Ovid) up to 26.05.20*

#	Searches	**Results**
1	(lynch* adj3 syndrome).tw.	6998
2	((lynch* adj3 famil*) and (cancer* or neoplasm*)).tw.	916
3	(Hereditary Nonpolyp* Colorectal Cancer or Hereditary Non-polyp* Colorectal Cancer).tw.	5441
4	HNPCC.tw.	5046
5	(((hereditary or inherit*) adj3 (colon* or colorectal* or ovar* or endometrial or endometrium or uterine or uterus)) and (cancer* or neoplasm*)).tw.	12689
6	((hereditary adj3 (nonpolyp* or non-polyp*)) and (colon* or colorectal*)).tw.	6921
7	((hereditary adj3 (cancer* or neoplasm*)) and (colon* or colorectal* or ovar* or endometrial or endometrium or endometrioid or uterine or uterus)).tw.	9290
8	((Famil* adj3 (Nonpolyp* or Non-polyp*)) and (colon* or colorectal*)).tw.	515
9	(famil* adj3 (colon* or colorectal* or ovar* or endometrial or endometrium or endometrioid or uterine or uterus)).tw.	11327
10	exp Colorectal Neoplasms, Hereditary Nonpolyposis/ or exp Ovarian Neoplasms/ or exp Ovary cancer/ or exp Ovary carcinoma/ or exp Endometrial Neoplasms/ or exp Endometrium cancer/ or exp Endometrioid carcinoma/ or exp Endometrium carcinoma/ or (exp Neoplasms/ and (exp Gastrointestinal disorders/ or exp Ovaries/ or exp Uterus/))	718392
11	((((microsatellite or micro-satellite) adj3 instabilit*) or (msi adj3 test*) or ((mismatch or mis-match) adj1 repair) or (mmr adj3 test*)) and (colon* or colorectal* or lynch* or ovar* or endometrial or endometrium or endometrioid or uterine or uterus)).tw.	18336
12	((amsterdam or bethesda) adj1 criteri*).tw.	1438

13	((EPCAM* or (MLH1 or hMLH1 or MSH2 or hMSH2 or MSH6 or hMSH6 or PMS2 or hPMS2)) and (colon* or colorectal* or lynch* or ovar* or endometrial or endometrium or endometrioid or uterine or uterus)).tw.	12045
14	(exp Genetic Predisposition to Disease/ and (exp colorectal cancer/ or exp Ovarian Neoplasms/ or exp Ovary cancer/ or exp Ovary carcinoma/ or exp Endometrial Neoplasms/ or exp Endometrium cancer/ or exp Endometrioid carcinoma/ or exp Endometrium carcinoma/)) or ((exp predisposition/ or exp genetics/ or exp "susceptibility (disorders)"/) and (exp gastrointestinal disorders/ or exp Ovaries/ or exp Uterus/))	20716
15	exp Adenomatous Polyposis Coli/	13582
16	(gardner syndrome or adenomatous polyp*).tw.	22822
17	Genes, APC/ and (colon* or colorectal*).tw.	8264
18	((MUTYH* or MYH*) and (colon* or colorectal*)).tw.	1399
19	((AFAP or FAP) and (colon* or colorectal*)).tw.	3867
20	Hamartoma Syndrome, Multiple/	2264
21	(Hamartoma adj3 (syndrome or cancer* or neoplas* or colon* or colorectal* or ovar* or endometrial or endometrium or endometrioid or uterine or uterus)).tw.	1096
22	((Bannayan-Riley-Ruvalcaba* or Cowden* or peutz-jegher* or peutz jegher* or juvenile polyp*) adj3 (syndrome or disease)).tw.	6884
23	((STK11 or SMAD4 or BMPR1A) and (colon* or colorectal*)).tw.	1695
24	(PTEN and (colon or colorectal or ovar* or endometrial or endometrium or endometrioid or uterine or uterus)).tw.	5728
25	Peutz-Jeghers Syndrome/	5267
26	Colonic Polyps/ or colon disorders/	16248
27	(exp Genes, BRCA1/ or exp BRCA1 Protein/ or exp Genes, BRCA2/ or exp BRCA2 Protein/) and (exp Ovarian Neoplasms/ or exp Ovary cancer/ or exp Ovary carcinoma/)	11165

28	(exp BRCA2 Protein/ or exp Genes, BRCA2/ or exp Genes, BRCA1/ or exp BRCA1 Protein/) and ovar*.tw.	12047
29	((Hereditary breast adj2 ovar* cancer*) or ((breast cancer adj3 gene*) and ovar*)).tw. or "Hereditary Breast and Ovarian Cancer Syndrome"/	4931
30	((HBOC or BRCA*) and ovar*).tw.	14909
31	1 or 2 or 3 or 4 or 5 or 6 or 7 or 8 or 9 or 10 or 11 or 12 or 13 or 14 or 15 or 16 or 17 or 18 or 19 or 20 or 21 or 22 or 23 or 24 or 25 or 26 or 27 or 28 or 29 or 30	788386
32	GENOMICS/ or genetics/ or genome/	935627
33	Genetic Testing/	75284
34	Genetic disorders/ and (exp gastrointestinal disorders/ or exp Ovaries/ or exp Uterus/)	223
35	Genetic Counseling/	43865
36	genetic counsel*.tw.	41430
37	((gene or genetic or genom* or multigene or multi-gene or genotyp*) adj3 (test* or panel*)).tw.	122058
38	((universal adj3 tumo?r) and screening).tw.	90
39	((germline* or germ-line*) adj3 test*).tw.	2026
40	Genetic Services/	1304
41	Genetic Carrier Screening/ or (Health screening/ and Genetic testing/)	15276
42	32 or 33 or 34 or 35 or 36 or 37 or 38 or 39 or 40 or 41	1144592
43	Health Plan Implementation/	96213
44	(implementation$ or disseminat$ or knowledge translation).tw.	813919

45	Translational Medical Research/ or "research and development"/	215310
46	(knowledge transfer or rollout$ or roll-out$ or treatment plan$ or care plan$ or innovation* or complex intervention or mainstream* or (pathway* adj3 (management or clinical or care or referral))).tw.	375861
47	"Delivery of Health Care"/ or Health Care Delivery/	260881
48	(health care servic* or health care utili?ation).tw.	57898
49	Hospitals, Public/ or Public Health Services/ or exp Health Care Services/	154226
50	Health Services Research/	66667
51	(clinical service* or hospital program*).tw.	15598
52	((facilitat* or challenge* or enabl* or change agent) adj3 health).tw.	48511
53	(organi?ation* adj2 (chang* or intervention* or modif*)).tw.	22306
54	(system* adj2 (chang* or intervention* or modif*)).tw.	69167
55	((practi?e* or practi?ing) adj2 chang*).tw.	29852
56	((program* adj2 (chang* or intervention* or modif* or introduc* or evaluat*)) or (test adj3 (uptake or access*))).tw.	124664
57	"Referral and Consultation"/ or professional referral/ or professional consultation/	146727
58	Organizational Innovation/ or innovation/	149738
59	"Delivery of Health Care, Integrated"/ or Health Care Utilization/	134420
60	(scal* up or referral*).tw.	308273
61	43 or 44 or 45 or 46 or 47 or 48 or 49 or 50 or 51 or 52 or 53 or 54 or 55 or 56 or 57 or 58 or 59 or 60	2671233

62	31 and 42 and 61	2184
63	remove duplicates from 62	1653
64	limit 63 to english language	1589
65	limit 64 to yr="1980 -Current"	1574
66	animals/ not humans/	5791706
67	65 not 66	1566
