# Supplementary material for: Health system interventions to integrate genetic testing in routine oncology services: A systematic review
Source: PLoS One. 2021 May 19;16(5):e0250379. doi: 10.1371/journal.pone.0250379 (PMC8133413; doi:10.1371/journal.pone.0250379)
Supplement: S2 Table — (PDF) [file pone.0250379.s002.pdf]

S2 Table. Search strategy CINAHL (EBSCO) up to 26.05.20*

Search ID	Search	Results
S1	TI (lynch* N3 syndrome) or AB (lynch* N3 syndrome)	532
S2	TI ((lynch* N3 famil*) and (cancer* or neoplasm*)) or AB ((lynch* N3 famil*) and (cancer* or neoplasm*))	59
S3	TI (Hereditary Nonpolyp* Colorectal Cancer or Hereditary Non-polyp* Colorectal Cancer) or AB (Hereditary Nonpolyp* Colorectal Cancer or Hereditary Non-polyp* Colorectal Cancer)	207
S4	TI (HNPCC) or AB (HNPCC)	138

S5	TI (((hereditary or inherit*) N3 (colon* or colorectal* or ovar* or endometrial or endometrium or uterine or uterus)) and (cancer* or neoplasm*)) or AB (((hereditary or inherit*) N3 (colon* or colorectal* ovar* or endometrial or endometrium or uterine or uterus)) and (cancer* or neoplasm*))	445
S6	TI ((hereditary N3 (nonpolyp* or non-polyp*)) and (colon* or colorectal*)) or AB ((hereditary N3 (nonpolyp* or non-polyp*)) and (colon* or colorectal*))	248
S7	TI ((hereditary N3 (cancer* or neoplasm*)) and (colon* or colorectal* or ovar* or endometrial or endometrium or endometrioid or uterine or uterus)) or AB ((hereditary N3 (cancer* or neoplasm*)) and (colon* or colorectal* or ovar* or endometrial or endometrium or endometrioid or uterine or uterus))	813
S8	TI ((Famil* N3 (Nonpolyp* or Non-polyp*)) and (colon* or colorectal*)) or AB ((Famil* N3 (Nonpolyp* or Non-polyp*)) and (colon* or colorectal*))	29
S9	TI (famil* N3 (colon* or colorectal* or ovar* or endometrial or endometrium or endometrioid or uterine or uterus)) or AB (famil* N3 (colon* or colorectal* or ovar* or endometrial or endometrium or endometrioid or uterine or uterus))	736
S10	(MH "Colorectal Neoplasms, Hereditary Nonpolyposis+") OR (MH "Ovarian Neoplasms+") OR (MH "Endometrial Neoplasms")	14,260
S11	TI ((((microsatellite or micro-satellite) N3 instabilit*) or (msi N3 test*) or ((mismatch or mis-match) N1 repair) or (mmr N3 test*)) and (colon* or colorectal* or lynch* or ovar* or endometrial or endometrium or endometrioid or uterine or uterus)) or AB ((((microsatellite or micro-satellite) N3 instabilit*) or (msi N3 test*) or ((mismatch or mis-match) N1 repair) or (mmr N3 test*)) and (colon* or colorectal* or lynch* or ovar* or endometrial or endometrium or endometrioid or uterine or uterus))	855
S12	TI ((amsterdam or bethesda) N1 criteri*) or AB ((amsterdam or bethesda) N1 criteri*)	71
S13	TI ((EPCAM* or (MLH1 or hMLH1 or MSH2 or hMSH2 or MSH6 or hMSH6 or PMS2 or hPMS2)) and (colon* or colorectal* or lynch* or ovar* or endometrial or endometrium or endometrioid or uterine or uterus)) or AB ((EPCAM* or (MLH1 or hMLH1 or MSH2 or hMSH2 or	386

	MSH6 or hMSH6 or PMS2 or hPMS2)) and (colon* or colorectal* or lynch* or ovar* or endometrial or endometrium or endometrioid or uterine or uterus))	
S14	(((MH “Disease Susceptibility”) OR (MH “Rare Diseases”)) AND ((MH "Intestinal Neoplasms+") OR (MH "Ovarian Neoplasms+") OR (MH "Endometrial Neoplasms"))	766
S15	(MH "Adenomatous Polyps+")	866
S16	TI (gardner syndrome or adenomatous polyp*) or AB (gardner syndrome or adenomatous polyp*)	874
S17	((MH "Genes, Neoplasm+") AND ((MH "Intestinal Neoplasms+") OR (MH "Ovarian Neoplasms+") OR (MH "Endometrial Neoplasms")))	64
S18	TI ((MUTYH* or MYH*) and (colon* or colorectal*)) or AB ((MUTYH* or MYH*) and (colon* or colorectal*))	53
S19	TI ((AFAP or FAP) and (colon* or colorectal*)) or AB ((AFAP or FAP) and (colon* or colorectal*))	112
S20	(MH "Hamartoma Syndrome, Multiple+")	147
S21	TI (Hamartoma N3 (syndrome or cancer* or neoplas* or colon* or coloretal* or ovar* or endometrial or endometrium or endometrioid or uterine or uterus)) or AB (Hamartoma N3 (syndrome or cancer* or neoplas* or colon* or coloretal* or ovar* or endometrial or endometrium or endometrioid or uterine or uterus))	86
S22	TI ((Bannayan-Riley-Ruvalcaba* or Cowden* or peutz-jegher* or peutz jegher* or juvenile polyp*) N3 (syndrome or disease)) or AB ((Bannayan-Riley-Ruvalcaba* or Cowden* or peutz-jegher* or peutz jegher* or juvenile polyp*) N3 (syndrome or disease))	332
S23	TI ((STK11 or SMAD4 or BMPR1A) and (colon* or colorectal*)) or AB ((STK11 or SMAD4 or BMPR1A) and (colon* or colorectal*))	47

S24	TI (PTEN and (colon or colorectal or ovar* or endometrial or endometrium or endometrioid or uterine or uterus)) or AB (PTEN* and (colon or colorectal or ovar* or endometrial or endometrium or endometrioid or uterine or uterus))	206
S25	((MH "Genes, BRCA") AND (MH "Ovarian Neoplasms+")	628
S26	TI ((MH "Genes, BRCA") AND (ovar*)) OR AB ((MH "Genes, BRCA") AND (ovar*))	541
S27	TI (Hereditary breast N2 ovar* cancer*) OR AB (Hereditary breast N2 ovar* cancer*)	342
S28	TI ((breast cancer N3 gene*) and (ovar*)) OR AB ((breast cancer N3 gene*) and (ovar*))	308
S29	TI ((HBOC or BRCA*) and (ovar*)) OR AB ((HBOC or BRCA*) and (ovar*))	1034
S30	(MH "Peutz-Jeghers Syndrome")	182
S31	(MH "Colonic Polyps")	1550
S32	(MH "Genetics+") OR (MH "Genetic Counseling")	147,487
S33	TI (genetic counsel*) or AB (genetic counsel*)	3,202
S34	TI ((gene or genetic or genom* or multigene or multi-gene or genotyp*) N3 (test* or panel*)) or AB ((gene or genetic or genom* or multigene or multi-gene or genotyp*) N3 (test* or panel*))	9661
S35	TI ((universal N3 (tumour or tumor)) and screening) or AB ((universal N3 (tumour or tumor)) and screening)	12
S36	TI ((germline* or germ-line*) N3 test*) or AB ((germline* or germ-line*) N3 test*)	161

S37	((MH “Evaluation Research+”) OR (MH “Program Development+”))	160,592
S38	TI (implementation* or disseminat* or knowledge translation) or AB (implementation* or disseminat* or knowledge translation)	95,483
S39	TI (knowledge transfer or rollout* or roll-out* or treatment plan* or care plan* or innovation* or complex intervention or mainstream*) or AB (knowledge transfer or rollout* or roll-out* or treatment plan* or care plan* or innovation* or complex intervention or mainstream*) or TI (pathway* N3 (management or clinical or care or referral)) or AB (pathway* N3 (management or clinical or care or referral))	68,087
S40	(MH "Health Care Delivery+")	277,631
S41	TI (health care servic* or health care utili?ation) or AB (health care servic* or health care utili?ation)	25,161
S42	(MH "Hospitals, Public+")	13,031
S43	(MH "Health Services Research+")	20,336
S44	TI (clinical service* or hospital program*) or AB (clinical service* or hospital program*)	14,479
S45	TI ((facilitat* or challenge* or enabl* or change agent) N3 health) or AB ((facilitat* or challenge* or enabl* or change agent) N3 health)	14,207
S46	TI (organi?ation* N2 (chang* or intervention* or modif*)) or AB (organi?ation* N2 (chang* or intervention* or modif*))	4272
S47	TI (system* N2 (chang* or intervention* or modif*)) or AB (system* N2 (chang* or intervention* or modif*))	9820
S48	TI ((practi?e* or practi?ing) N2 chang*) or AB ((practi?e* or practi?ing) N2 chang*)	9737

S49	TI (program* N2 (chang* or intervention* or modif*or introduc*)) or AB (program* N2 (chang* or intervention* or modif* or introduc*)) or TI (test N3 (uptake or access*)) or AB (test N3 (uptake or access*))	16,648
S50	(MH "Referral and Consultation+")	33,898
S51	(MH "Diffusion of Innovation")	11,773
S52	(MH "Health Care Delivery, Integrated")	8941
S53	TI (scal* up or referral*) or AB (scal* up or referral*)	41,194
S54	S1 OR S2 OR S3 OR S4 OR S5 OR S6 OR S7 OR S8 OR S9 OR S10 OR S11 OR S12 OR S13 OR S14 OR S15 OR S16 OR S17 OR S18 OR S19 OR S20 OR S21 OR S22 OR S23 OR S24 OR S25 OR S26 OR S27 OR S28 OR S29 OR S30 OR S31	19,391
S55	S32 OR S33 OR S34 OR S35 OR S36	152,600
S56	S37 OR S38 OR S39 OR S40 OR S41 OR S42 OR S43 OR S44 OR S45 OR S46 OR S47 OR S48 OR S49 OR S50 OR S51 OR S52 OR S53	671,204
S57	S54 AND S55 AND S56	439
S58	S54 AND S55 AND S56<br><br>Limiters - English Language; Published Date: 19800101-20181031	439

*An alert was set up for both searches outlined in Table 1 and 2 with 910 alerts included for the final 2915 records identified through database searching
