# Supplementary material for: Health system interventions to integrate genetic testing in routine oncology services: A systematic review
Source: PLoS One. 2021 May 19;16(5):e0250379. doi: 10.1371/journal.pone.0250379 (PMC8133413; doi:10.1371/journal.pone.0250379)
Supplement: S3 Table — (PDF) [file pone.0250379.s003.pdf]

S3 Table. Inclusion and exclusion criteria for routine genetic testing integration intervention studies in oncology

Selection criteria	Inclusion criteria	Exclusion criteria
**Study type**	Interventions	
**Study design**	Randomised control trials (RCTs) – including step wedge and cluster RCT<br>Non-randomised quasi-experimental design<br>-Cohort study<br>-Before and after study (including<br>interrupted time series and multiple baseline design)<br>Observational studies<br>-cohort studies<br>-Case series for intervention outcomes only<br>Qualitative-<br>Qualitative studies that report on implementation outcomes	Case reports, case series, case-control, cross-sectional, designed with no comparator<br>Cross-sectional: Single point in time knowledge study no before or after (no comparison)<br>Case series (no comparator) except if report on an intervention outcome<br>Qualitative studies if they do not report on implementation or intervention outcomes
**Population**	Health providers of genetic testing and/or counselling for HBOC/LS including (but not limited to);<br>- genetic counsellors,<br>- clinical geneticists,<br>- oncologists<br>FOR<br>Adult patients (>18 years old) diagnosed with the following cancers;<br>- ovarian<br>- breast<br>- colorectal<br>- endometrial	Health providers with no involvement in mainstreaming genetic testing<br>Patients with other cancers not related to hereditary cancer syndromes HBOC and hereditary colorectal cancer LS<br>No specific data for the subgroup of interest<br>Paediatric cancer patients <18 years<br>Asymptomatic individuals at high risk of HBOC and LS<br>Asymptomatic relatives of HBOC and LS identified families

	Minimum of 80% of population has to have the above cancers	
**<u>Intervention</u>**	Interventions aiming to implement pre-test genetic counselling and genetic or genomic testing through mainstreaming* for breast and ovarian cancer<br><br><u>OR</u><br><br>Interventions to increase pre-test genetic counselling and genetic testing completion rates after universal tumour screening (UTS) for colorectal and endometrial cancer.<br><br><u>For example, through increasing;</u><br><br>- the knowledge/awareness of health providers re HBOC or LS<br><br>- patient access to genetic testing<br><br>- identification of hereditary cancer such as HBOC and LS<br><br>- follow up of HBOC/LS patients getting through the health system<br><br><u>OR</u><br><br>Multicomponent interventions that target the health provider and patient to achieve the above	Interventions not used to increase identification of HBOC or LS<br><br>Research genetic or genomic testing<br><br>Laboratory methods of genetic testing<br><br>Data on likelihood of HBOC/LS mutation detection, mutation incidence or phenotype without any information on mainstreaming of genetic testing for the patient or health provider<br><br>Childhood-onset hereditary cancer<br><br>Multi component interventions aimed<br><br>solely at the patient except if the patient intervention is targeted to influence the health system<br><br>Studies with UTS steps not involved in mainstreaming<br><br>Physician discretionary referral to genetic counselling
**Comparator**	Standard care/no intervention<br><br>Another Intervention	
**Outcomes**	*<u>Implementation Outcomes</u>*–<br><br>-Acceptability<br><br>-Adoption<br><br>-Appropriateness<br><br>-Feasibility<br><br>-Cost<br><br>-Fidelity<br><br>-Penetration<br><br>-Sustainability	Outcomes not linked to mainstreaming of genetic or genomic testing or enhancing the uptake of universal tumour screening to improve identification of HBOC and LS

	<u>Service Outcomes</u><br>-Efficiency<br>-Safety<br>-Effectiveness<br>-Equity<br>-Patient centeredness<br>-Timeliness<br>*<u>Client outcomes</u>*<br>-Satisfaction<br>-Function<br>-Symptomatology<br>**<u>CFIR</u>**<br>-Intervention Characteristics<br>-Inner Setting<br>-Outer Setting<br>-Characteristics of Individuals<br>-Process	
**Language**	English	Not in English
**Publication period**	From January 1st 1980 - present	Before 1980
**Publication type**	Journal article	Conference proceedings, posters, comments or editorials, letters, news, editorials, narrative reviews, theses, review

- For the purposes of this systematic review mainstreaming is the process where all patients with a particular cancer are offered direct access to genetic testing in oncology care through pre-test genetic counselling regardless of who does the genetic counselling (eg. could be specialist or genetic counsellor).
