# Supplementary material for: Health system interventions to integrate genetic testing in routine oncology services: A systematic review
Source: PLoS One. 2021 May 19;16(5):e0250379. doi: 10.1371/journal.pone.0250379 (PMC8133413; doi:10.1371/journal.pone.0250379)
Supplement: S4 Table — (PDF) [file pone.0250379.s004.pdf]

S4 Table. Complex interventions to increase genetic counselling, testing and identification of hereditary in ovarian, breast, colorectal and endometrial cancer

Reference	Implementation strategies and framework	Participants and Health Setting	Intervention Influence	Framework mapping	Quality and method
Complex interventions to increase genetic counselling, testing and identification of hereditary ovarian and breast cancer					
Uyar et al [36]<br><br>2018<br><br>USA	*Content:*<br><br>I: Step wise process change to increase access to genetic testing (GT) and that genetic counselling (GC) for all women with epithelial ovarian cancer (EOC) using electronic health record (EHR), education, GC apt scheduling and team meeting (n=125 EOC patients)<br><br>C: Standard referral pathway to GC from oncologist or surgeon (n=207 EOC patients)<br><br>*Duration:*<br>**I:** Dec 2013–Nov 2016<br>C: Jan 2008–Nov 2013<br><br>*Implementation framework:*<br>Process change model	*Healthcare Professionals:*<br><br>All gynaecology oncology providers non-specified<br><br>*Healthcare Institution:*<br><br>Academic cancer centre	1. Rates of GC/GT recommendation in EHR<br><br>I: 110/125 (88%)<br>C:42/207 (20.3%)<br>Absolute difference (diff) + 67.7% (95% CI 59.8-75.6)<br>(no stats)<br><br>2.GC referral<br><br>I: 122/125 (97.6%)<br>C: 96/207 (46.4%)<br>Absolute diff +51.2% (95% CI 43.9-58.5)<br>p ≤0.001<br><br>3.GC completion<br><br>I: 108/125 (86.4%)<br>C:67/207 (32.4%)<br>Absolute diff +54% (95% CI 45.3 – 62.8)<br>p ≤0.001<br><br>GT completion<br><br>I:103/108 (95.4%)<br>C: 55/67 (82%)<br>Absolute diff +13.2% (95%CI 3.3 – 23.3) p=0.007<br><br>4.Patients identified with mutations	*Service:*<br><u>Effectiveness</u><br>-GC referral<br>-GC completion<br>-GT completion<br>-Patients with identified gene mutations<br><br><u>Equity</u><br>-GT access<br>-GC referrals<br>-GT undertaken<br><br>*Client:*<br><u>Cancer prevention</u><br>-Identification of hereditary Cancer<br><br>**CFIR**<br>*Inner setting*<br><u>Readiness for implementation</u><br>-access to knowledge and information<br><br>*Process*<br><u>Engaging</u><br>– key stakeholders<br><br><u>Executing</u>	Fair Quality Cohort study with historical control<br><br>Single site health system and no analysis on confounding variables or regression analysis on the characteristics inherent in the control verses the intervention population or health system

**Reference**	**Implementation strategies and framework**	**Participants and Health Setting**	**Intervention Influence**	**Framework mapping**	**Quality and method**
			I:22/101 (21.8%)<br>C: 10/55 (18.2%)<br>Absolute diff = +3.6% (95% CI -9.4 – 16.5) p=0.68		
Kentwell et al[33]<br><br>2017<br><br>Australia	*Content:*<br><br>I: Embedded a GC as a member of the onsite gynaecology oncology team for pre-test GC during chemotherapy chair time or after an oncology appointment. A specialized referral form incorporating current Australian genetic testing guidelines for EOC and referral triage at a weekly multidisciplinary meeting.<br>-An introduction education session for all referring specialists about integrating the new referral pathway. (n= 190 EOC patients)<br><br>C: Standard referral pathway to GC from oncologist or surgeon<br>(n= 212 EOC patients)<br><br>*Duration:*<br>**I:** June 2014 - May 2016<br>C: June 2010–May 2013<br><br>*Implementation framework:*<br>NR	*Healthcare Professionals*<br><br>Gynaecology oncologist<br>Specialist nurse<br>Medical oncologist<br>Genetic Counsellor<br><br>*Healthcare Institution:*<br><br>Publically funded Cancer unit at a major treating centre –cancer genetics services available	1. GC referral<br><br>I: 129/152 (85%)<br>C: 73/134 (54%)<br>Absolute difference = +30.4% (95% CI 20.2-40.6) p≤0.001<br><br>2. Time to gain access to GC and results<br>Referral to GC<br>I:2014-15 - 42 days<br>2015-16- 54.5 days<br><br>Referral to results<br>2014-15 - 106 days<br>2015-16- 140.5 days<br>C: NR<br><br>3. Patients identified with mutations<br>I: 2014-2015<br>7/34 (20.6%)<br>2015-2016<br>4/30 (13.3%)<br>C: NR<br><br>4.Familial predictive GT uptake<br>I:31/120 (28%)<br>C:NR<br><br>5. A high level of comfort with the process of consenting to and delivering results for genetic testing amongst the medical oncologists (n	*Implementation:*<br>Acceptability<br>-Satisfaction with mainstreaming<br><br>*Service:*<br>Efficiency<br>-Time to gain access to GT and results<br>Effectiveness<br>-GC referral<br>-Patients with identified gene mutations<br>Equity<br>-GT access<br>-GC referral<br><br>*Client:*<br>Cancer prevention<br>-Identification of hereditary Cancer<br><br>**CFIR**<br>*Inner setting*<br>Readiness for implementation<br>-access to knowledge and information<br>- available resources	Poor Quality Case series with no control<br><br>Single site health system

Reference	Implementation strategies and framework	Participants and Health Setting	Intervention Influence	Framework mapping	Quality and method
			= 6), but less so amongst the gynaecology oncologists (n = 4) and medical oncology trainees (n = 1)	*Process*<br><u>Engaging</u><br>– key stakeholders<br><br>*Characteristics of Individuals*<br>Self-efficacy	
George et al[29]<br><br>2016<br><br>UK	*Content:*<br><br>I: A mainstreaming implementation toolkit with online education in pre-test GC and *BRCA* testing protocol, information sheet for patients with cancer who receive a normal, mutation or a variant of unknown significance, consent form for testing and frequently asked questions for breast and gynaecology health professionals (n= 207 EOC patients)<br><br>C: Standard referral pathway to GC from oncologist or surgeon<br>(n= NR EOC patients) data not presented<br><br>*Duration:*<br>**I:** July 2013 – Nov 2014<br>C: NR<br><br>*Implementation framework:*<br>NR	*Healthcare Professionals*<br><br>Gynaecology oncologist<br>Specialist nurse<br>Medical oncologist<br>Genetic Counsellor<br><br>*Healthcare Institution:*<br><br>Publically funded Cancer unit at a major treating centre – cancer genetics services available	1. GC referral<br><br>I: 207/207 (100%)<br>C: NR<br><br>2.Time to gain access to genetic test results<br>4-fold reduction in time to result<br><br>3.Patients identified with mutations<br><br>I: 33/207 (16%)<br>C: NR<br><br>4.Treatment management<br>I:132/207 (64%) 20/23 BRCA+ - PARPi access<br>C: NR<br>I: 31/32 (97%) with mutations breast cancer surveillance<br>C: NR<br>5. Satisfaction and comfort with mainstreaming intervention<br>I:105/105 (100%) patients were pleased to have had the genetic test<br>15/15 (100%) clinicians were comfortable with consenting for genetic testing<br>C: NR	*Implementation:*<br><u>Acceptability</u><br>-Satisfaction with mainstreaming intervention<br><br><u>Cost</u><br>-implementation cost<br><br>*Service:*<br><u>Efficiency</u><br>**-**Time to gain access to GT<br><br><u>Effectiveness</u><br>-GC referral<br>-Patients with identified gene mutations<br><br>*<u>Equity</u>*<br>-GT access<br>-GC referral<br><br><u>Patient centeredness</u><br>**-**Patients satisfaction with	Poor Quality Case series with no comparator to control<br><br>Single site health system

Reference	Implementation strategies and framework	Participants and Health Setting	Intervention Influence	Framework mapping	Quality and method
			6. Cost<br>13-fold reduction in genetics appointments with annual cost saving of 2.6 million	mainstreaming intervention<br><br>*Client:*<br><u>Cancer prevention</u><br>-Identification of hereditary Cancer<br>-Access to cancer prevention information<br>-Referral for cancer prevention<br><br>**CFIR**<br>*Intervention Characteristics*<br>- Cost<br><br>*Inner setting*<br><u>Readiness for implementation</u><br>-access to knowledge and information<br><br>*Process*<br><u>Engaging</u><br>– key stakeholders<br><br>*Characteristics of Individuals*<br>Self-efficacy	
Swanson et al [37]<br><br>2018	*Content:*<br>I: Provider education (quarterly resident/fellow training) on national recommendations for GC referral, information	*Healthcare Professionals:*<br><br>Surgeon	1. GC referral<br><br>I:40/56 (71.4%)<br>C: 33/75 (44.0%)	*Service:*<br><u>Effectiveness</u><br>-GC referral	Fair Quality<br>Cohort study with historical control

Reference	Implementation strategies and framework	Participants and Health Setting	Intervention Influence	Framework mapping	Quality and method
USA	regarding genetic testing, the goals of the quality improvement project, and components of the intervention toolkit (checklist for EOC patients in outpatient gynaecology clinic, referral recommendations for GC in patients hospital, electronic order for outpatient GC prior to hospital discharge and coordination of GC appointment with the patient's 6- week postoperative examination appointment and mailed the patient a family cancer history worksheet prior (n= 62 EOC patients)<br><br>C: Standard referral pathway to GC from oncologist or surgeon<br>(n= 81 EOC patients)<br><br>*Duration:*<br>**I:** July 2015 – December 2015<br>C: July 2013–December 2013<br><br>*Implementation framework:*<br>DMAIC (Define, Measure, Analyze, Improve, Control) Methodology	Allied health staff<br>Nurse<br>Administrative<br>Resident and fellow, Medical oncologist<br>Geneticist<br>Genetic counsellors<br><br>*Healthcare Institution;*<br><br>A tertiary care centre – offsite GC practice	Absolute difference = +27.4% (95% CI 11.1-43.7) p=0.02<br><br>2. GC completion<br><br>I: 24/40 (60%)<br>C: 29/33 (87.9%)<br>Absolute difference = -27.8% (95% CI -46.7 to -9.1) (no stats)<br><br>GT Completion<br><br>I: 24/24 (100%)<br>C: 23/29; 79.3%<br>Absolute difference = +20.6 (95% CI 5.9-35.4) (no stats)<br><br>3.Patients identified with mutations<br><br>I: 3/24; 12.5%<br>C: 7/23; 30.4%<br>Absolute difference = - 17.9 (95% CI – 40.9 – 5.1) p=0.17	-GC and GT completion<br><br>Equity<br>-GT access<br>-GC referral<br>-GT undertaken<br><br>*Client:*<br>Cancer prevention<br>-Identification of hereditary Cancer<br><br>**CFIR**<br>*Inner setting*<br>Readiness for implementation<br>-access to knowledge and information<br><br>*Process*<br>Engaging<br>– key stakeholders<br><br>Executing	Single site health system and no analysis on confounding variables or regression analysis on the characteristics inherent in the control verses the intervention population or health system
Senter et al [34]<br><br>2017<br><br>USA	*Content:*<br>I: Genetics embedded model (GEM) with a licensed GC embedded in the outpatient Gynaecologic Oncology (GO) clinic on two full days per week in one of two clinic locations. A GC referral through EMR and GO staff scheduled the GC appointment to co-inside with GO follow-up visits or treatments (e.g. chemotherapy infusion visits) (n= 336 EOC patients)	*Healthcare Professionals:*<br><br>Gynaecology oncology and cancer genetics health professionals-unspecified	1.GC referral<br><br>I:147/336 (44%)<br>C:84/401 (21%)<br>Absolute difference = +22.8% (95% CI 16.7 – 29.4) p<0.00001<br><br>2.GC completion<br><br>I:123/147 (84%)<br>C:32/84 (38%)	*Service:*<br>Effectiveness<br>-GC referral<br>-GC and GT completion<br><br>*Equity*<br>-GT access<br>-GC referrals<br>-GT undertaken	Good quality Cohort study with historical control

Reference	Implementation strategies and framework	Participants and Health Setting	Intervention Influence	Framework mapping	Quality and method
	C: Standard referral pathway to GC from oncologist or surgeon to cancer genetics services off-site (n= 401 EOC patients)<br><br>*Duration:*<br>**I:** August 2014–September 2016<br>C: Nov 2011- July 2014<br><br>*Implementation framework:*<br>NR	*Healthcare Institution;*<br><br>Large academic medical comprehensive cancer centre with off-site GC until 08/2014	Absolute difference = +45.5% (95% CI 33.6 – 57.6) p<0.00001<br><br>GT completion<br><br>I:97%<br>C:96%<br><br>3. Time to gain access to GC<br><br>I: 1.67 months<br>C:2.52 months<br>P< 0.01	Timeliness<br>-Time to GC apt<br><br>**CFIR**<br>*Inner setting*<br>Readiness for implementation<br>-available resources<br><br>*Process*<br>Engaging<br>– key stakeholders	
Tutty et al[48]<br><br>2019<br><br>Australia	*Content:*<br><br>I: Telephone Genetic counselling (TGC) care pathway where the oncologists refer EOC patients to TGC for pre-test GC over the telephone by GC.<br>Patient receives forms and test kit via post and has blood drawn locally. Genetic test results received and reviewed with a Geneticist and GC provides post-test TGC, informs referring oncologist and organises follow up in person counselling for positive results and those with a family history (n=284 EOC patients)<br><br>C: Those with EOC who had standard in person GC (SIGC)<br>(n= 52 EOC patients)<br><br>*Duration:*<br>I: January 2016 - May 2017<br>C: January 2008 – December 2013<br><br>*Implementation framework:*<br>NR	*Healthcare Professionals:*<br><br>Genetic counsellors<br>Geneticist<br>Gynaecologic oncologist<br><br>*Healthcare Institution:*<br><br>Metropolitan Australian Familial Cancer Centre	1.GC referral<br><br>I: 284<br>C: NR<br>No stats<br><br>2.GC and GT completion<br><br>I: 284<br>C: NR<br>No stats<br><br>3.Patients identified with mutations<br><br>I: 26/284 (9%)<br>C: NR<br>No stats<br><br>4. Acceptability and cost of intervention<br>Acceptability<br>I:97.2% and 94.3% were satisfied with the timing of the telephone call and information provided (n=107)	*Implementation:*<br>Acceptability<br>-Satisfaction with TGC intervention<br><br>Cost<br>-Implementation cost<br><br>*Service:*<br>Efficiency<br>-Cost of Resources to implement the intervention<br><br>Effectiveness<br>-GC referral and completion rate<br>-GT completion<br>-Patients with identified gene mutations	Poor Quality<br>Case series with no comparator to control<br><br>Single site health system

Reference	Implementation strategies and framework	Participants and Health Setting	Intervention Influence	Framework mapping	Quality and method
			C: NR<br>Cost<br>I: $91.52 per woman tested (n=72)<br>C: $ 107. 37 SIGC (n=52)<br>Absolute diff cost-saving - $15.85	Equity<br>-GT access<br>-GC referral<br><br>Patient centeredness<br>-Patients satisfaction with TGC intervention<br><br>**CFIR**<br>*Intervention Characteristics*<br>- Cost<br><br>*Outer setting*<br>Needs & Resources of Those Served by the Organization<br><br>*Process*<br>Engaging<br>– key stakeholders	
Bednar et al[35]<br><br>2017<br><br>USA	*Content:*<br><br>I: Physician-coordinated genetic testing (PCGT) via gynaecologic oncologist (GO) to performed GC - consent for genetic testing and sample collection, completed all paperwork, and disclosed the results to the patient<br>**OR**<br>Integrated genetic counselling (IGC) via GC integrated into the gynaecologic oncology clinic and tumour board conferences – 2.5 GC prioritized appointments for EOC	*Healthcare Professionals:*<br><br>Physicians<br>Genetic counsellors<br>Advanced practice providers<br>Nurses	1-2. PCGT or IGC<br><br>I:561/1214 (46.2%) main campus clinic<br>PCGT 84/151 (55.6%) regional clinic<br>653/1214 (53.8%) outside institution<br>C: NR No stats	*Service:*<br>Effectiveness<br>- GT undertaken<br>- GC referral<br>- GC apt uptake<br><br>Equity<br>-GT access<br>-GC referral<br>-GT undertaken	Poor Quality<br>Case series with no comparator to control

Reference	Implementation strategies and framework	Participants and Health Setting	Intervention Influence	Framework mapping	Quality and method
	patients and group GC email address used for urgent or same-day appointment requests<br>**OR**<br>Assisted genetic counselling referral (AGCR) a patient tracking system to document recommendations for GC and GT- Patients without documentation of GC or GT were identified through patient tracking.<br>A GC referral was made in EMR system and email notification to the GO physician re referral and signature. GC appointment was schedule as per standard procedures. (n= 1214 EOC patients)<br><br>C: NR<br><br>*Duration:*<br>I: 2013 - 2015<br>C: NR<br><br>*Implementation framework:*<br>Plan-Do-Study-Act cycle method	Clinical managers<br>Physician trainees<br><br>*Healthcare Institution:*<br><br>An Academic Cancer Center's (regional and main campus clinics)	3. AGCR<br><br>I: 33/34 (97%) signed GC electronic referrals<br>14/72 (19.4%) email referrals<br><br>C: NR No stats<br>4. GT completed<br><br>I: 1214/1423 (85.3%)<br>C: NR No stats<br><br>5. Patients identified with *BRCA* mutations<br><br>I: 217/1214 (17.9%)<br>C: NR No stats<br><br>6. Time to gain access to GC<br><br>I: 78 days by 2015<br>C: 197 days in 2012<br>Absolute difference 119 days	<u>Timeliness</u><br>-Time to GC apt<br><br>Cl*ient:*<br><u>Cancer prevention</u><br>- Identification of hereditary Cancer<br><br>**CFIR**<br>*Inner setting*<br><u>Readiness for implementation</u><br>-available resources<br><br>*Process*<br><u>Engaging</u><br>– key stakeholders<br><br><u>Executing</u>	
Hanley et al[45]<br><br>2018<br><br>Canada	*Content:*<br><br>I: A Province wide educational campaign to increase awareness of cancer prevention through referral of ovarian cancer patients for GC & GT and inclusion of recommendation regarding the importance of referral into pathology reports (n = 426 ovarian cancer)<br><br>C: Usual care with no education on recommendations as above (n = 456 ovarian cancer)<br><br>*Duration:*<br>I: 2010 -2013<br>C: 2001 – 2010	*Healthcare Professionals:*<br><br>Family practitioners<br>General obstetrician<br>Gynaecologists<br>Medical and gynaecologic oncologists<br><br><br>*Healthcare Institution:*	1. GC and GT completion<br><br>Serous<br>I:311/426 (72.5%)<br>C: 270/456 (59.0&)<br>Absolute difference +13.7% (95% CI 7.6 -19.1)<br>(OR = 4.70; 95% CI 2.89–7.62)<br><br>Endometrioid<br>I: 29/426 (6.8%)<br>C: 60/456 (13.1%)<br>Absolute difference = -6.3% (95% CI -6.4 to – 2.4)	*Service:*<br><u>Effectiveness</u><br>- GT undertaken<br>- GC uptake<br><br><u>Equity</u><br>-GT access<br>-GT undertaken<br><br>*Client:*<br><u>Cancer prevention</u><br>- Identification of hereditary Cancer	Fair to poor quality<br>Cohort study with historical control<br><br>Multisite health system but with no analysis on confounding variables or regression analysis on the characteristics

Reference	Implementation strategies and framework	Participants and Health Setting	Intervention Influence	Framework mapping	Quality and method
	Implementation framework:<br>NR	State wide Hereditary cancer program	Clear cell<br>I: 15/426 (3.5%)<br>C: 31/456 (6.8%)<br>Absolute difference = -3.3% (95% CI -6.2 to -0.4)<br><br>Unknown<br>I: 71/426 (16.6%)<br>C: 95/456 (20.7%)<br>Absolute difference = -4.2<br>P< 0.001 serous vs endometroid and clear cell cancers getting GT after 2010<br><br>2. Patients identified with *BRCA* mutations<br><br>Serous<br>I: 48/60 (79.7%)<br>C: 76/103 (73.8%)<br>Absolute difference = +6.2% (95% CI -6.1 to 19.4) P=0.519<br><br>3.Familial predictive GT uptake and mutation identification<br>Carrier tests per BRCA positive ovarian cancer patient<br>I: 3.27<br>C:2.54<br>Absolute difference +0.73 p=0.071<br><br>Family members identified as BRCA positive<br>I: 2.18<br>C: 1.62<br>Absolute difference +0.56 p= 0.009<br><br>Carrier tests per BRCA positive serous cancer patient	**CFIR**<br>*Inner setting*<br>Readiness for implementation<br>- access to knowledge and information<br><br>*Process*<br>Engaging<br>– key stakeholders	inherent in the control verses the intervention population or health system<br><br>Unclear how many patients were followed up

Reference	Implementation strategies and framework	Participants and Health Setting	Intervention Influence	Framework mapping	Quality and method
			I: 3.36<br>C: 2.60<br>Absolute difference + 0.76 P=0.098<br><br>Family members identified as BRCA positive<br>I: 2.29<br>C: 1.64<br><br>Absolute difference +0.65 P=0.012		
Petzel et al[46]<br><br>2014<br><br>USA	*Content:*<br><br>I: Referral form within the electronic medical record (EMR), including a 1-page summary of genetic referral guidelines for ovarian cancer in a checklist format, allowing oncologists to systematically and automatically refer directly to the Cancer Genetics Clinic (n = 83)<br><br>C: Standard referral without use of EMR referral (n=86)<br><br>*Duration:*<br>I: May 2008 – May /2009<br>C: May 2007 – May 2008<br><br>*Implementation framework:*<br>NR	*Healthcare Professionals:*<br><br>Gynaecologic oncologists<br>Genetic Counsellor<br><br>*Healthcare Institution:*<br><br>Primary academic metro Women's Cancer Centre	1.GC referral<br><br>I: 25/83 (30.12%)<br>C:15/86 (17.44%)<br>Absolute difference = +12.7% (95% CI -0.04 – 25.4) P=0.053<br><br>2.GC completion<br><br>I:16/83 (19.3%)<br>C:8/86 (9.3%)<br>Absolute difference = +9.9% (95% CI – 0.41 – 20.4)	*Service:*<br>Effectiveness<br>- GC referrals<br>- GC uptake<br><br>Equity<br>-GT access<br>-GC referral<br>-GT undertaken<br><br>**CFIR**<br>*Inner setting*<br>Readiness for implementation<br>- access to knowledge and information<br><br>*Process*<br>Engaging<br>– key stakeholders	Good quality Cohort study with historical control<br><br>Single site with regression analysis on the characteristics inherent in the control verses the intervention population or health system but no analysis on confounding variables
Cohen et al[43]	*Content:*	*Healthcare Professionals:*	1.GC referral<br><br>I:75/145 (51.7%)	*Service:*<br>Effectiveness<br>- GC referral	Fair Quality

Reference	Implementation strategies and framework	Participants and Health Setting	Intervention Influence	Framework mapping	Quality and method
2016<br><br>Australia	I: Genetics attendance at a gynaecologic oncology tumour board meeting (n = 145 EOC)<br><br>C: No attendance of genetics at gynaecologic oncology tumour board meeting (n = 116 EOC)<br><br>*Duration:*<br>I: July 2014 -June 2015<br>C: July 2013 - June 2014<br><br>*Implementation framework:*<br>NR	Geneticist<br>Genetic Counsellor<br>Oncologists<br><br><br>*Healthcare Institution:*<br><br>Metropolitan hospital	C:31/116 (26.7%)<br>Absolute difference = +25% (95% CI 13.6 – 36.4) (P < 0.0001).<br><br>2. GC completion<br><br>I: 67/75 (89%)<br>C: 30/31 (96%)<br>Absolute difference = -7.4% (95% CI – 16.8 to 1.9)<br><br>GT completion<br><br>I:47/67 (70%)<br>C:26/30 (86.6%)<br>Absolute difference = -16.5% (95% CI -32.9 tp – 0.14)<br><br>3.Patients identified with *BRCA* mutations<br><br>I:16/75 (21%)<br>C:6/31 (19%)<br>Absolute difference = +1.9% (95% CI -22.9 – 26.9)	- GT undertaken<br><br>Equity<br>-GT access<br>-GC referral<br>-GT undertaken<br><br><br>*Client:*<br>Cancer prevention<br>- Identification of hereditary Cancer<br><br>**CFIR**<br>*Process*<br>Engaging<br>– key stakeholders	Cohort study with historical control<br><br>State-wide health system with no analysis on confounding variables or regression analysis on the characteristics inherent in the control verses the intervention population or health system
Percival et al[30]<br><br>2016<br><br>UK	*Content:*<br><br>Online education for pre-test GC and written material (the protocol to identify patients,<br>information leaflet on *BRCA* testing, information on significance of a normal, mutation or VUS *BRCA* result and familial implications) delivered by the genetics team.<br>Checklist and self-certification of competency for nurses completed before consenting patients for *BRCA* testing (n = 108 EOC)<br><br>C: Consent for BRCA performed by doctor (n = 192 EOC)<br><br>*Duration:*	*Healthcare Professionals:*<br><br>Clinical Nurse specialist in oncology<br>Medical Oncologists<br><br>*Healthcare Institution:*<br><br>Single centre metro hospital	1. Patient satisfaction with pre-test GC<br><br>I: 108/300 (36%) Nurse<br>C: 192/300 (64%) Doctor<br>No difference in patient satisfaction between those consented by a nurse or a doctor<br><br>I: 75/108 (69%) patients consented by nurses completed a questionnaire.	*Implementation:*<br>Acceptability<br>-Satisfaction with mainstreaming intervention<br><br>*Client:*<br>Patients satisfaction with mainstreaming intervention<br><br>**CFIR**<br>*Inner setting*	Poor quality Case series with no comparator to control<br><br>Single site health system

Reference	Implementation strategies and framework	Participants and Health Setting	Intervention Influence	Framework mapping	Quality and method
	I: July 2013-December 2015<br>C: July 2013-December 2015		No patients refused GT, or requested a GC appointment before GT.<br>C: NR<br><br>Nurses satisfaction with pre-test GC training and role<br><br>I: 5/6 (83%) nurses found the BRCA training helpful and saw BRCA testing was part of their role and felt supported.<br>C: NR	Readiness for implementation<br>- access to knowledge and information<br><br>*Outer setting*<br>Needs & Resources of Those Served by the Organization<br><br>*Characteristics of Individuals*<br>Self-efficacy<br><br>*Process*<br>Engaging<br>– key stakeholders	
Rahman et al[32]<br><br>2017<br><br>UK	*Content:*<br><br>I: Mainstream genetic testing pathway giving direct access to GT in oncology clinics. Online Education via the Marsden Mainstreamed Genetic Testing in Cancer Programme[29]<br><br>*Duration:*<br>I: February 2015 - April 2016<br>C: NR<br><br>*Implementation framework:*<br>NR	*Healthcare Professionals:*<br>Medical/clinical oncologists<br><br>*Healthcare Institution:*<br><br>Tertiary oncology centre	1.GT completion<br><br>I: 122/NR<br>C: NR<br>No stats<br><br>2.Patients identified with BRCA mutations<br><br>I: 18/122 (14.8%)<br>C: NR<br>No stats<br><br>3.Time to gain access to GT, results & GC referral	*Service:*<br>Effectiveness<br>- GT undertaken<br><br>*Equity*<br>-GT access<br>-GT undertaken<br><br>Timeliness<br>-Time to access GT, results and GC referral<br><br>*Client:*<br>Cancer prevention	Poor Quality Case series with no comparator to control<br><br>Single site health system

Reference	Implementation strategies and framework	Participants and Health Setting	Intervention Influence	Framework mapping	Quality and method
			I: The time from sample receipt to result was between 14–48 working days --GC referral between 12 - 43 working days after MGT results<br>-20/56 (36) had MGT within 1 month of diagnosis<br>C: NR<br>No stats<br><br>4. Treatment management impact<br><br>I: 11/18 (67%) no change in management<br>6/18 (33%) access PARPi<br>C: NR<br>No stats<br><br>5.Familial predictive GT uptake<br><br>I: 11/ 15 (73%) family members of BRCA carriers having predictive GT<br>C: NR<br>No stats	- Identification of hereditary Cancer<br><br>**CFIR**<br>*Inner setting*<br>Readiness for implementation<br>- access to knowledge and information<br><br>*Process*<br>Engaging<br>– key stakeholders	
Plaskoinska et al[31]<br><br>2016<br><br>UK	*Content:*<br><br>I: Pre-test GC by information leaflet and telephone by a study co-ordinator through a designed mainstreaming pathway with post-test GC for BRCA positive or VUS in genetic services (n = 232 EOC)<br><br>*C:*NR<br><br>*Duration:*<br>I: July 2013 – June 2015<br>C: NR<br><br>*Implementation framework:*<br>NR	*Healthcare Professionals:*<br><br>Genetic Counsellor<br>Oncologist<br>Study co-ordinator<br><br>*Healthcare Institution:*<br><br>Rural and metro publically funded hospitals of	1.GT completion<br><br>I: 232/281 (83%)<br>C: NR<br>No stats<br><br>2. Patients identified with BRCA mutations<br><br>I: 18/232 (8%)<br>C: NR<br>No stats<br><br>3. Time to gain access to genetic test results	*Implementation:*<br>Acceptability<br>-Satisfaction with mainstreaming intervention<br>Cost<br>-Implementation cost<br><br>*Service:*<br>Effectiveness<br>- GC referral<br>- GT undertaken<br><br>Equity	Poor Quality<br>Case series with no comparator to control

Reference	Implementation strategies and framework	Participants and Health Setting	Intervention Influence	Framework mapping	Quality and method
		different sizes, ranging from smaller district general hospitals to large regional centres	I: Consent to results delivery 46 working days<br>C: NR<br>No stats<br><br>4.Acceptability and feasibility of mainstreaming pathway<br>Acceptability<br><br>I: 173/232 (75%) low psychological impact to GT compared to cancer diagnosis ($p<0.001$).<br>C: NR<br><br>I: 174/232 (75%) had enough information and time to decide to have GT<br>C: NR<br><br>Cost<br><br>I: £121 229 mainstreaming pathway<br>C:£130 102 current standard pathway	-GT access<br>-GC referrals<br>-GT undertaken<br><br>Efficiency<br>-Time to gain access to GT results<br><br>Patient centeredness<br>-Patients satisfaction with mainstreaming intervention<br><br>*Client:*<br>Cancer prevention<br>- Identification of hereditary Cancer<br><br>**CFIR**<br>*Intervention Characteristics*<br>- Cost<br><br>*Outer setting*<br>Needs & Resources of Those Served by the Organization<br><br>*Process*<br>Engaging<br>– key stakeholders	

Reference	Implementation strategies and framework	Participants and Health Setting	Intervention Influence	Framework mapping	Quality and method
Bednar et al[39]<br><br>2019<br><br>USA	*Content:*<br><br>I: Step wise process change to increase access to GT and GC for all women with EOC using IGC, electronic health record (EHR), education, GC apt scheduling and team meeting<br>*IGC started on 6/30/2015;*<br>• Integrate GC in Gynaecology Oncology clinic<br>• Optimize GC schedule and standardized urgent appointment<br>(N=33 ovarian cancer).<br>*Physician education started on 12/1/2015*<br>• Physicians attend national meetings and conferences discussing hereditary cancer.<br>• GC provide education as needed.<br>Clinic patient tracking started on 1/1/2016<br>• Research data coordinator collected data from clinic schedules and the medical record to determine whether patients received GC/GT.<br>(n=14 ovarian cancer)<br>AGCR started on 1/1/2016<br>• Electronic referral to GC drafted for patients who have not had GC/GT (N=110)<br>Provider email notifications *started on 1/1/2016*<br>• Research data coordinator and GC notify physician/care team of upcoming patients not previously referred for GC/GT.<br><br>(N = 57 ovarian cancer)<br><br>C: Usual care for referral to GC for ovarian cancer (N = NR)<br><br>*Duration:*<br>I: June 2015 – August 2017<br>C: Prior to June 2015<br><br>*Implementation framework:*	*Healthcare Professionals:*<br><br>Genetic counsellor<br>Gynaecologic oncologists<br>Nurses<br>Advanced practice registered nurses (APRN)<br><br>*Healthcare Institution:*<br><br>Regional hospital – single site with a gynaecologic oncology clinic	1.GC referral<br><br>I: 48/57 (84.2%)<br>C: NR<br>(p = 0.02)<br><br>2. GC and GT completion<br><br>I: 43/48 (89.6%) completed GC<br>39/43(90.7%) completed GT<br>C: NR (p = 0.03)<br><br>3. Patients identified with mutations<br><br>I: 8/39 (20.5%)<br>C: NR<br>No stats	*Service:*<br>Effectiveness<br>- GT undertaken<br>- GC referral<br>- GC apt uptake<br>- TT undertaken<br><br>*Equity*<br>-GT access<br>-GC referrals<br>-GT/TT undertaken<br><br><br>*Client:*<br>Cancer prevention<br>- Identification of hereditary Cancer<br><br>**CFIR**<br>*Inner setting*<br>Readiness for implementation<br>- access to knowledge & information<br>-available resources<br><br>*Process*<br>Engaging<br>– key stakeholders<br><br>Executing	Poor Quality Case series with no comparator to control<br><br>Single site health system

Reference	Implementation strategies and framework	Participants and Health Setting	Intervention Influence	Framework mapping	Quality and method
	Model for Improvement quality improvement framework includes Plan-Do Study-Act (PDSA) cycles				
Brown et al[38]<br><br>2018<br><br>USA	*Content:*<br><br>I: Patient navigators in the gynaecologic oncology clinics reviewed all patients seen and identified patients meeting GT criteria and facilitated GC via in-person or telegenetics consultations.<br>Education was provided to all gynaecologic oncologists and advanced care providers on guidelines in departmental and research meetings<br>Referral to GC was made a standard of practice and GC staff increase from one to eight.<br><br>(n= 332 EOC)<br><br>C: NR<br><br>*Duration:*<br>I: 2013-2015<br>C: NR<br><br>*Implementation framework:*<br>NR	*Healthcare Professionals*<br>Gynaecologic oncologists<br>Breast surgeons<br>Genetic counsellors<br>Patient navigators<br>Advanced care providers<br><br>*Healthcare Institution:*<br><br>Comprehensive not-for-profit system with more than 900 care locations in 2 states,<br>and 16 rural locations with GC services via 6 in-person clinics and telemedicine at 5 sites.	1. GC referral<br><br>I: 107/111 (96%)<br>C: 41/112 (37%)<br>Absolute difference = +59.7% (95% CI 50.2 – 69.4) p<0.05<br><br>2. GT completion<br><br>I: 59/111 (53%)<br>C:27/112 (24%)<br>Absolute difference = +29% (95% CI 16.8 -41.2) p<0.05<br><br>3. Patients identified with BRCA mutations<br><br>I:11/59 (19%)<br>C:3/27 (11%)<br>Absolute difference = +7.5% (95% CI – 7.9 – 23) p = 0.53	*Service:*<br>*Effectiveness*<br>- GT undertaken<br>- GC referrals<br>*Equity*<br>-GT access<br>-GC referrals<br>-GT undertaken<br><br>*Client:*<br>Cancer prevention<br>- Identification of hereditary Cancer<br><br>**CFIR**<br>*Inner setting*<br>Readiness for implementation<br>- access to knowledge & information<br>-available resources<br><br>*Process*<br>Engaging<br>– key stakeholders	Poor Quality<br>Case series with no comparator to control
Richardson et al[54]<br><br>2020	*Content:*<br><br>Oncology clinic-based GT. Direct access to pre-test GC and panel GT through oncologists in a population based	*Healthcare Professionals:*<br>Oncologists<br>Genetic counsellor	1. Acceptability<br>I: Patients indicated comfort and acceptability with the GT process - no difference between oncology	*Implementation:*<br>Acceptability<br>-Satisfaction with mainstreaming intervention	Good to Fair quality

Reference	Implementation strategies and framework	Participants and Health Setting	Intervention Influence	Framework mapping	Quality and method
Canada	program through an oncology clinic-based model with post-test counselling provided by a genetic counsellor<br>Oncologists were;<br><br>-trained by one of the hereditary cancer program (HCP) medical directors to provide GT information to patients and consent patients for GT.<br><br>-provided with a frequently asked questions (FAQ) information sheet, a standardized script outline and patient consent form with continued HCP support.<br>(n = 49, breast and ovarian cancer patients)<br><br>C: The traditional model of referral to GC services (n = 99, breast and ovarian cancer patients)<br><br>*Study Duration:*<br>I: August 2015–July 2017<br>C: August 2015–July 2017<br><br>*Implementation framework:*<br>NR	*Healthcare Institution:*<br><br>Population state based cancer program in Canada	clinic-based model (OCB) and the traditional model (TM).<br>OCB M = 4.54, SD = 0.71 vs TM M = 4.52, SD = 0.69<br>8/19 oncologists completed survey – 5/8 strongly agreed or agreed with 'the process for carrying out multi-gene panel testing worked well', Knowledge, MICRA and DCS scores - participants produced no significant in scores between the TM and OCB models.<br>C: NR<br><br>2. GC completion<br>I: OCB 49/49 (100%) and 24/49 (48.9 %) in person<br>4/49 (8.2 %) by telephone<br>21/49 (42.9 %) by videoconference<br><br>C: TM 41/99 (41.4%) and 8/99 (8.1 %) in person<br>24/99 (24.2) and 91/99 (91.9) by telephone<br>34/99 (34.3) by videoconference<br><br>Absolute difference +58.6 % (95% CI 49-68) and +8.5 % (95% CI -8.2 - 25) in person and videoconference<br>P< 0.001 OCB vs TM<br><br>3. GT completion<br>I: OCB (14 gene panel) - 21/49 (42.9%)<br>(17 gene panel) - 28/49 (57.1%)<br>C: TM (14 gene panel) - 34/99 (34.3 %)<br>(17 gene panel) - 49/99 (49.5 %)	*Service:*<br>Effectiveness<br>- GT undertaken<br>- GC referral<br>Equity<br>-GT access<br>-GC referral<br>-GT undertaken<br><br>*Client:*<br>Knowledge<br>Acceptability<br>Satisfaction<br>Cancer prevention<br>- Identification of hereditary Cancer<br><br>**CFIR**<br>*Inner setting*<br>Readiness for implementation<br>- access to knowledge & information<br>-available resources<br><br>*Outer setting*<br>Needs & Resources of Those Served by the Organization<br><br>*Process*<br>Engaging<br>– key stakeholders	Cohort study with concurrent control<br><br>State-wide health system with analysis on confounding variables or regression analysis on the characteristics inherent in the control verses the intervention population or health system<br><br>Representation of patient population selective – all patients didn't complete survey. Small proportion of all patients included

Reference	Implementation strategies and framework	Participants and Health Setting	Intervention Influence	Framework mapping	Quality and method
			Absolute difference +8.5 % (95% CI -8.2 -25) and 7.6% (95% CI -9.4 – 25) p = 0.015 OCB vs TM<br><br>4. Patients identified with *BRCA* mutations<br>I: 5/49 (10.2 %)<br>C: 7/99 (7.1%)<br><br>Absolute difference +3.1 % (95% CI -6.7 - 13) p = 0.507 OCB vs TM<br><br>5. Time to gain access to GT results<br>I: M = 191 days, SD 174<br>C: M=403 days, SD =312<br><br>Absolute difference -212 days P< 0.001 OCB vs TM	Reflecting & Evaluating	
Rumford et al[52]<br><br>2020<br><br>United Kingdom	*Content:*<br><br>I: Mainstream genetic testing pathway giving direct access to GT in oncology clinics. Online Education via the Marsden Mainstreamed Genetic Testing in Cancer Programme[29]<br><br>The first 32 patients tested were consented following a group consenting process via a lecture on *BRCA* testing and then offered a consultation and blood test the same day (n= 255, ovarian cancer patients)<br><br>C: *NR*<br><br>*Duration:*<br>I: April 2016 – April 2018<br>C: NR	*Healthcare Professionals:*<br>All gynaecology oncology health professionals<br><br>*Healthcare Institution:*<br><br>Imperial College NHS Trust Imperial College Hospital Mainstreaming Programme (ICHMP).	1. GC referral<br><br>I:255/268 95%<br>C: NR no stats<br><br>2. GC and GT completion<br><br>I: 255/268 95%<br>C: NR no stats<br><br>3. Patients identified with *BRCA* mutations<br>I:34/255; 13.3%<br>C: NR no stats<br><br>4: Time to gain access to GT	*Service:*<br>Efficiency<br>-Time to gain access to GT<br>*Effectiveness*<br>-GC referral<br>-GC completion<br>-GT completion<br><br>*Client:*<br>Equity<br>-GT access<br>-GC referral<br>-GT undertaken<br>Cancer prevention	Poor Quality<br>Case series with no comparator to control<br><br>Single site health system

Reference	Implementation strategies and framework	Participants and Health Setting	Intervention Influence	Framework mapping	Quality and method
	Implementation framework:<br>NR		I: Turnaround time between blood sample and return of GT result was 20.6 (11–42) calendar days<br>C: Turnaround time of 148.2 calendar days prior to I<br>No stats<br><br>5. Treatment management impact<br>I: 9/34 received a PARPi<br>5/34 receiving platinum-based chemotherapy – clinician intent to initiate PARPi chemotherapy<br>15/34 still receiving first-line (adjuvant) treatment or in remission - not eligible for PARPi<br>5/34 ineligible to receive PARPi<br>C: NR no stats	-Identification of hereditary Cancer<br><br>**CFIR**<br>*Inner setting*<br>Readiness for implementation<br>- access to knowledge & information<br>-available resources<br><br>*Process*<br>Engaging<br>– key stakeholders	
McLeavy et al[51]<br><br>2020<br><br>UK	I: Mainstream genetic testing pathway giving direct access to GT in oncology clinics. Online Education via the Marsden Mainstreamed Genetic Testing in Cancer Programme[29] (n=170 EOC)	*Healthcare Professionals:*<br>Oncologist<br><br>*Patients:*<br>Ovarian cancer patients<br><br>*Healthcare Institution:*<br>Publically funded tertiary referral centre	1. Acceptability<br>I: Decision Regret Scale 9.14±12.397 - 14/29 (48.3%), reported no decision regret<br>26/29 (89.6%) were satisfied with their decision to pursue GT<br>Participants produced relatively low MICRA scores regardless of mutation status<br>C: NR<br><br>2. GC completion<br>I:170 (100)<br>C: NR no stats<br><br>3. GT completion<br>I:170 (100)<br>C: NR no stats<br><br>4. Patients identified with *BRCA* mutations<br>I:23/170 (13.5)	*Implementation:*<br>*Acceptability*<br>-Satisfaction with decision to undergo GT<br><br>*Service:*<br>*Effectiveness*<br>-GT completed<br>-Patients with identified gene mutations<br>*Patient centeredness*<br>-Patients satisfaction with mainstreaming intervention<br><br>*Equity*<br>-GT access<br>-GT undertaken	Poor Quality<br>Case series with no comparator to control<br><br>Single site tertiary hospital setting

Reference	Implementation strategies and framework	Participants and Health Setting	Intervention Influence	Framework mapping	Quality and method
			C:NR no stats	*Client:*<br>*<u>Cancer prevention</u>*<br>-Identification of hereditary Cancer<br><br>**CFIR**<br>*Inner setting*<br><u>Readiness for implementation</u><br><u>-</u> access to knowledge & information<br><br>*Outer setting*<br><u>Needs & Resources of Those Served by the Organization</u><br><br>*Process*<br><u>Engaging</u><br>– key stakeholders	
Kemp et al[40]<br><br>2019<br><br>UK	*Content:*<br><br>I: A mainstreaming implementation toolkit with online education in pre-test GC with certificate of completion and multicomponent toolkit for oncology health professional including; Breast cancer BRCA testing protocol, information sheets on BRCA testing and result outcome normal, mutation or VUS information sheets for patients, consent for genetic testing and frequently asked questions for breast and gynaecology clinicians re BRCA testing (n= 1184 breast cancer patients)<br><br>C: *NR*<br>*Duration:*	*Healthcare Professionals:*<br><br>All gynaecology oncology and cancer genetics health professionals unspecified<br><br>*Healthcare Institution:*	1. GT completion<br><br>I: 1184/1184 (100%)<br>C: NR No stats<br><br>2. Patients identified with *BRCA* mutations<br>I: 117/1184 (9.9%)<br>C: NR<br><br>3. GC completion after GT<br>I: 115/117 (98.3)<br>C:NR	*Implementation<u>:</u>*<br>*<u>Acceptability</u>*<br>-Satisfaction with mainstreaming intervention<br><br>*Service:*<br>*<u>Effectiveness</u>*<br>-GT completion<br>-Patients with identified gene mutations<br>*<u>Patient centeredness</u>*	Poor Quality Case series with no comparator to control<br><br>Single site health system

Reference	Implementation strategies and framework	Participants and Health Setting	Intervention Influence	Framework mapping	Quality and method
	I: September 2013 - February 2017<br>C: NR<br><br>*Implementation framework:*<br>Quality improvement program	Publicly funded cancer unit at a major treating centre – cancer genetics services available	4. Acceptability of mainstreaming process<br>I: 129/259 (50%) patients surveyed 128/128 (100%) -pleased to have GT 124/129 (96.1%) -happy that GT was via cancer team.<br>23/23 (100%) of cancer team members reported<br>feeling confident to do BRCA testing during their consultation and believed that the process worked well<br><br>C: NR<br><br>5. Feasibility<br>I: 2,500 genetics appointments<br>C: 50,000 genetics appointments<br>95% reduction in genetic consultation<br>85% reduction in time to test result compared with traditional approach<br>Discounted QALY of 2746 compared to no testing	-Patients satisfaction with mainstreaming intervention<br><br>*Equity*<br>-GT access<br>-GT undertaken<br><br>*Client:*<br>*Cancer prevention*<br>-Identification of hereditary Cancer<br><br>**CFIR**<br>*Intervention Characteristics*<br>- Cost<br><br>*Inner setting*<br>Readiness for implementation<br>- access to knowledge & information<br><br>*Outer setting*<br>Needs & Resources of Those Served by the Organization<br><br>*Characteristics of Individuals*<br>Self-efficacy<br><br>*Process*<br>Engaging	

Reference	Implementation strategies and framework	Participants and Health Setting	Intervention Influence	Framework mapping	Quality and method
				– key stakeholders<br><br>Reflecting & Evaluating<br><br>Executing	
Brown et al[38]<br><br>2018<br><br>USA	*Content:*<br>I: Patient navigators in the breast surgery clinics reviewed all patients seen and identified patients meeting GT criteria and facilitated GC via in-person or telegenetics consultations. Education was provided to all breast surgeons, and advanced care providers on guidelines in departmental and research meetings.<br>Referral to GC was made a standard of practice and GC staff increase from one to eight.<br>(n= 313 TNBC < 60years<br>and 664 BrCa < 45 years)<br><br>C: NR<br><br>*Duration:*<br>I: 2013-2015<br>C: NR<br><br>*Implementation framework:*<br>Not recorded	*Healthcare Professionals*<br><br>Gynaecology oncologists<br>Breast surgeons<br>Genetic counsellors<br>Patient navigators<br>Advanced care providers<br><br>*Healthcare Institution:*<br><br>Comprehensive not-for-profit system with more than 900 care locations in 2 states,<br>and 16 rural locations with GC services via 6 in-person clinics and telemedicine at 5 sites.	1. GC referral<br>TNBC < 60 yrs<br>I: 107/118 (91%)<br>C: 66/95 (69%)<br>Absolute difference = +21.2% (95% CI 10.6 – 31.8) p<0.05<br>BrCa < 45 yrs<br>I:193/228 (85%)<br>C: 163/208 (78%)<br>Absolute difference = +6.3 % (95% CI -1.0 – 13.5)<br><br>2. GT completion<br><br>TNBC < 60 yrs<br>I: 101/118 (86%)<br>C:56/95 (59%)<br>Absolute difference = +26.6 % (95% CI 14.9 – 38.4) p<0.05<br>BrCa < 45 yrs<br>I: 186/228 (82%)<br>C:137/208 (66%)<br>Absolute difference = +15.7% (95% CI -7.5 – 6.1) p<0.05<br><br>3. Patients identified with BRCA mutations<br>TNBC < 60 yrs<br>I:13/101(13%)<br>C:6/56 (10.7%)<br>Absolute difference = +0.22% (95% CI -8.2 -12.6)	*Service:*<br>*Effectiveness*<br>- GT undertaken<br>- GC referral<br>*Equity*<br>-GT access<br>-GC referral<br>-GT undertaken<br><br>*Client:*<br>Cancer prevention<br>- Identification of hereditary Cancer<br><br>**CFIR**<br>*Inner setting*<br>Readiness for implementation<br>- access to knowledge & information<br>-available resources<br><br>*Process*<br>Engaging<br>– key stakeholders	Poor Quality Case series with no comparator to control

Reference	Implementation strategies and framework	Participants and Health Setting	Intervention Influence	Framework mapping	Quality and method
			<u>BrCa < 45 yrs</u><br>I:18/186(10%)<br>C:14/137(10%)<br>Absolute difference = -0.54% (95% CI -7.2 -6.1)		
Lobo et al[55]<br><br>2018<br><br>Spain	*Content:*<br><br>A MDT hereofamilial cancer unit (HFCU) to assess breast cancer patients hereditary risk<br>• Specific hereditary cancer consultation by a medical oncologist with specific training in genetics<br>• Preparation of a pre and post-test report: discussion about genetic testing, post-test results (pathogenic, no pathogenic, and VUS) and risk reduction strategies/ surveillance.<br>• Centralization of blood draw in cancer nursing<br>• Referral to gynaecology-oncology to assess the risk reduction strategies after result disclosure<br>• Referral to psycho-oncology for identified patients<br>• Weekly heredofamilial cancer committee (comprised of the medical oncologist in charge of the HFCU, a gynaecologist with specific training in risk-reduction strategies, an oncology nurse, a psychologist, and a general surgeon) to discuss complex cases<br>(n = 832, breast cancer)<br><br>C: Usual care and referral pathway to a genetics unit<br>(n = 751, breast cancer)<br><br>*Duration:*<br>I: July 2010- June 2013<br>C: July 2007 – June 2010<br><br>*Implementation framework:*<br>NR	*Healthcare Professionals*<br>Medical oncologist<br>Cancer Nurse<br>Psychologist<br>General Surgeon<br>Gynaecologist<br><br>*Healthcare Institution:*<br>Hospital General Universitario Gregorio Marañón, Madrid Spain	1.Eligible for GC referral<br>I: 223/832 (26.8%)<br>C: 194/751 (25.8)<br>Absolute difference = +0.97% (95% CI -3.3 – 5.3)<br><br>2.GC referral<br>I: 114/223 (51.1%)<br>C: 50/194 (25.8%)<br>Absolute difference = +25.4% (95% CI 16.4 – 34.3) p < 0.0001<br><br>3.GT completion<br>I: 125/168 (74.4%)<br>C: 43/50 (86%)<br>Absolute difference = -11% (95% CI -23.3 – 0.069)<br><br>4.Patients identified with *BRCA* mutations<br>I: 17/125 (13.6%).<br>C: 8/43 (18.6%)<br>Absolute difference -5% (95% CI -18 – 8)<br><br>5.Cancer prevention management impact<br>I:8/17 (47%)<br>C:2/8 (25%)	*Service*<br><u>Effectiveness</u><br>-GC referral<br>-GC completion<br>-GT completion<br><br>*Client*<br><u>Equity</u><br>-GT access<br>GC referral<br><u>Cancer prevention</u><br>-Identification of hereditary Cancer<br>- cancer prevention strategies up taken<br><br>**CFIR**<br>*Inner setting*<br><u>Readiness for implementation</u><br>-available resources<br><br>*Process*<br><u>Engaging</u><br>– key stakeholders	Fair Quality<br>Cohort study with historical control<br><br>Single site health system and no analysis on confounding variables or regression analysis on the characteristics inherent in the control verses the intervention population or health system<br>Unclear how many patients followed up

Reference	Implementation strategies and framework	Participants and Health Setting	Intervention Influence	Framework mapping	Quality and method
			Absolute difference = +22% (95% CI -16.2 – 60.3) p=0.03		
Grinedal et al[53]<br><br>2020<br><br>Norway	*Content:*<br><br>GC and *BRCA* testing only offered through the treating oncologist and surgeon to eligible breast cancer patients with the use of;<br><br>• Genetics team developed written information and consent forms<br>• Genetics led informational meetings at all hospitals.<br>• Patients with a pathogenic variant or a VUS referred to GC.<br>• The patient's family history of cancer to be recorded on admission for treatment and normal *BRCA* results with a family history of cancer that indicated further GT referred to GC<br>**(**n**=** 361, breast cancer)<br><br>C: Usual care and referral pathway to a genetics department<br>(n = NR)<br><br>*Duration:*<br>I: January 2016 – June 2016<br>January 2017 - June 2017<br>C: NR<br><br>*Implementation framework:*<br>NR	*Healthcare Professionals:*<br>Medical oncologist<br>General Surgeon<br>Gynaecologist<br>Genetic Counsellor<br>Geneticist<br><br>*Healthcare Institution:*<br><br>Regional and urban hospital in Norway	1. GC referral<br><br>I:131/356 (36.8%)<br>C: NR no stats<br><br>2. GC completion<br><br>I:125/356 (34.6%)<br>C: NR no stats<br><br>Outcome 3. GT completed<br>I:125/131 (95.4%)<br>C: NR no stats	*Service:*<br><u>Effectiveness</u><br>-GC referral<br>-GC completion<br>-GT completion<br><br>*Client:*<br><u>Equity</u><br>- GT access<br>-GC referral<br>-GT undertaken<br><br>**CFIR**<br>*Inner setting*<br><u>Readiness for implementation</u><br><u>-</u> access to knowledge & information<br>-available resources<br><br>*Process*<br><u>Engaging</u><br>– key stakeholders	Poor Quality Case series with no comparator to control
Complex interventions to increase genetic counselling, testing and identification of hereditary colorectal and endometrial cancer					
Heald et al[44]<br><br>2013<br><br>USA	*Content:*<br><br>I: Pathologist and GC facilitation for systematic pre-test GC referral (n=1,108 CRC).<br><u>GC facilitated</u>	*Healthcare Professionals:*<br><br>Genetic Counsellor	1. GC referral<br><br>I: GC:56/56 (100.0%)<br>GC & Surgeon: 9/11 (81.8%)<br>C: No GC: 21/38 (55.3%)	*Service*:<br><u>Effectiveness</u><br>- GT undertaken<br>- GC referral<br>- GC apt uptake	Fair Quality Cohort study with historical control

Reference	Implementation strategies and framework	Participants and Health Setting	Intervention Influence	Framework mapping	Quality and method
	• MSI/IHC results to colorectal surgeon via pathology report in EMR<br>• MSI/IHC results (all) to GC weekly via email from pathologist<br>• Results disclosure by GC via telephone or letter on surgeon's behalf + facilitated GC referral<br>(n = 784 CRC)<br><u>GC facilitation & Surgeon communication</u><br>• MSI/IHC results to colorectal surgeon via pathology report in EMR<br>• MSI/IHC results (all) to GC weekly via email from pathologist<br>• GC notifies colorectal surgeon via email re: patients appropriate for GC referral<br>4. Results disclosure by surgeon + facilitates GC referral (n=87 CRC)<br><br>*C:* Usual care – <u>No GC facilitation</u><br>• MSI/IHC results to colorectal surgeon only via pathology report in EMR<br>• Results disclosure +/-referral at discretion of surgeon (n = 237<br>CRC)<br><br>*Duration:*<br>***I:*** GC facilitated:<br>July 2008-January 2012<br>GC & Surgeon facilitated:<br>August 2007-June 2008<br><br>*C:* No GC:<br>January 2004-July 2007<br><br>*Implementation framework:*<br>NR	Colorectal Surgeon Pathologist<br><br>*Healthcare Institution:*<br><br>Academic and tertiary (2 regional community hospitals) and primary care centres (multiple family health centres)	Absolute Difference =<br>GC v No GC +44.7% (95% CI 28.1 – 60.5) p<0.001<br>GC & Surgeon v No GC &Surgeon +26.5% (95% CI -1.2 – 54.2) p=0.023<br><br>2. GC completion<br>I: GC:40/56 (71.4%)<br>GC & Surgeon: 7/11 (63.6%)<br>C: No GC & Surgeon :12/38 (31.6%)<br><br>Absolute Difference =<br>GC v No GC +39.8% (95% CI 20.9 – 58.8) p<0.001<br>GC & Surgeon v No GC & Surgeon +32.0% (95% CI 0.017 – 64)<br>No stats<br><br>3. GT completion<br>I: GC:37/56 (66.1%)<br>GC & Surgeon: 5/11 (45.5%)<br><br>C: Surgeon & No GC: 10/38 (26.3%)<br><br>Absolute Difference =<br>GC v No GC +39.8% (95% CI 21.1 – 58.5) p<0.001<br>GC & Surgeon v No GC +19.2% (95% CI -13.4 – 51.7)<br>No stats<br><br>4. Patients identified with mutations<br>I: GC: 17/56 (30.4%)<br>GC & Surgeon: 1/11 (9.1%)<br>C: Surgeon & No GC: 3/38 (7.9%)<br><br>Absolute Difference =	<u>Timeliness</u><br>-Time to GC apt<br><br>*<u>Equity</u>*<br>-GT access<br>-GC referral<br>-GT undertaken<br><br>*Client:*<br><u>Cancer prevention</u><br>- Identification of hereditary Cancer<br><br>**CFIR**<br>*Inner setting*<br><u>Readiness for implementation</u><br>-available resources<br><br>*Process*<br><u>Engaging</u><br>– key stakeholders	Single site health system with no analysis on confounding variables or regression analysis on the characteristics inherent in the control verses the intervention population or health system<br><br>Less than 80% of population followed up

Reference	Implementation strategies and framework	Participants and Health Setting	Intervention Influence	Framework mapping	Quality and method
			GC v No GC +22.5% (95% CI 7.7 – 37.2)<br>GC & Surgeon v No GC +1.2% (95% CI -17.8 – 20.2)<br>No stats<br><br>5. Time to appointment<br>I: GC: 44 days<br>Surgeon & GC : 293 days<br><br>GC: 17/56 (30.4%)<br>GC & Surgeon<br>C: Surgeon & no GC : 457 days<br>Absolute Difference =<br>GC v Surgeon & GC -249 daysGC v Surgeon and No GC -413 days p<0.001<br>Surgeon & GC v Surgeon and No GC -164 days		
Cohen et al[47]<br><br>2016<br><br>USA	*Content:*<br>I: UTS with embedded GC and role delineation of OHP<br>• Clinic nurse tracked results and shared with all providers.<br>• A shared GC email inbox for medical genetics review of all results.<br>• Abnormal MSI/IHC results triggered an automatic GC referral<br>•Synchronous GC and colorectal clinic appointment scheduling.<br>(n= 44 CRC patients)<br><br>C: Usual care referral to GC at discretion of surgeon (n= 30 patients)<br><br>*Duration:*<br>I: July 2013-Dec 2013<br>*C:* Feb 2013-June 2013<br><br>*Implementation framework:*<br>NR	*Healthcare Professionals:*<br><br>Medical Oncology<br>Gastroenterology<br>Surgery<br>Pathology<br>Laboratory<br>Medical Genetics<br>Genetic Counselling<br><br>*Healthcare Institution:*<br><br>An outpatient cancer care centre for oncology patients	1. GC referral<br><br>I: 10/44 (22%)<br>C: 4/30 (13.3%)<br>Absolute Difference = +9.4% (95% CI -7.9 – 26.8)<br>2.GC completion<br><br>I: 10/44 (22.7%)<br>C: 4/30 (13.3%)<br>Absolute Difference = +9.4% (95% CI -7.9 – 26.8)<br><br>3. GT completion<br><br>I: 6/10 (60%)<br>C: 2/4 (50%)<br>Absolute Difference = +10% (95% CI -47.6 – 67.6)	*Service:*<br>Effectiveness<br>- GT undertaken<br>- GC apt uptake<br>Equity<br>- GT access<br>- GT undertaken<br><br><br>**CFIR**<br>*Inner setting*<br>Readiness for implementation<br>-available resources<br><br>*Process*<br>Engaging<br>– key stakeholders	Poor Quality Cohort study with historical control<br><br>Single site health system with no analysis on confounding variables or regression analysis on the characteristics inherent in the control verses the intervention population or health system

Reference	Implementation strategies and framework	Participants and Health Setting	Intervention Influence	Framework mapping	Quality and method
		treated at a tertiary academic National Cancer Institute (NCI)-designated Comprehensive Cancer Consortium			
Long et al[42]<br><br>2018<br><br>Australia	*Content:*<br>I: Electronic or e-mail referral form<br>Reminders for reflex BRAF testing<br>Checklist and follow up included in MDT proforma for GI<br>Education and information handouts on new genetics referral process to surgical and oncology with feedback on audit results of IHC testing and referral<br>Standardised text in pathology reporting and information sheets on how to interpret<br><br>(n= 203 CRC patients)<br><br>C: Baseline pathology and genetics referral before intervention design and initiation<br>(n= 184 CRC patients)<br><br>*Duration:*<br>I: February 2016 -November 2016<br>*C:* May 2014–April 2015<br><br>*Implementation framework:* Theoretical Domains Framework Implementation (TDFI) approach	*Healthcare Professionals:*<br><br>Medical oncologist<br>Surgeons<br>Pathologist<br>Genetic Counsellor and Geneticist<br>Radiation oncologist<br>Oncology nurses<br>Oncology and genetics admin<br>Palliative care<br><br>*Healthcare Institution:*<br>NR	1. Eligible for GC<br><br>I: Hospital A 11/77 (14%)<br>Hospital B 11/126 (8.7%)<br>C: Hospital A 5/71(7%)<br>Hospital B 12/113 (11%)<br><br>Absolute Difference =<br>Hospital A +7.24% (95% CI -2.3 -17)<br>Hospital B -1.88% (95% CI -9.4-5.6)<br><br>2. GC referral<br><br>I: Hospital A 6/11 (55%)<br>Hospital B 1/11 (9%)<br>C: Hospital A 4/5(80%)<br>Hospital B 1/12 (8.3%)<br><br>Absolute Difference =<br>Hospital A -25% (95% CI -71-20)<br>Hospital B +0.76% (95% CI -22-24)	*Service:*<br>*Effectiveness*<br>-GC referral<br><br>**CFIR**<br>*Inner setting*<br>Readiness for implementation<br>- access to knowledge & information<br>-available resources<br><br>*Process*<br>Engaging<br>– key stakeholders<br>Reflecting and evaluating	Poor Quality Cohort study with historical control<br><br>Two hospital sites but with no analysis on confounding variables or regression analysis on the characteristics inherent in the control verses the intervention population or health system
Miesfeldt et al[41]<br><br>2018	*Content:*<br>I: Screen-positive UTS results were communicated by phone or email from the pathologist to the treating surgeon, the patient navigator (PN) or both.	*Healthcare Professionals:*<br>Pathologist<br>Surgeon	1. GC referral<br><br>I: 16/16 (100.0%)<br>C:12/12 (100.0%)<br>No stats	*Service:*<br>*Effectiveness*<br>-GT undertaken<br>-GC referral<br>-GC apt uptake	Poor quality Case series with no comparator for control

Reference	Implementation strategies and framework	Participants and Health Setting	Intervention Influence	Framework mapping	Quality and method
USA	PN helped to coordinate referrals for genetic counselling and consideration of testing.<br><br>(n= 16 patients)<br><br>C: Non-navigated patients usual care –referral at the discretion of the surgeon<br>(n= 12 patients)<br><br>*Duration:*<br>**I:** May 2015 - April 2016<br>C: May 2015 -April 2016<br><br>*Implementation framework:*<br>NR	Patient navigator<br>- Oncology Nurse<br><br>*Healthcare Institution:*<br>Medical Centre Cancer Institute's Cancer Risk and Prevention Clinic - community hospital and a state tertiary centre with a GC-supported cancer genetic program<br><br>*Patients:*<br>All colorectal and uterine cancer	2. GC completion<br><br>I: 14/16 (87.5%)<br>C: 5/12 (41.7%)<br>Absolute Difference = +45.8% (95% CI 13.6 – 78.1) p=0.020<br><br>3. GT completion<br><br>I: 13/14 (92.9%)<br>C: 4/5 (80.0%)<br>Absolute Difference =<br>+12.9% (95% CI -24.7 – 50.4)<br><br>4. Patients identified with mutations<br><br>I: 7/13 (53.8%)<br>C: 1/4 (25.0%)<br>Absolute Difference =<br>+28.8% (95% CI -21.5 -79.2)	*Equity*<br>-GT access<br>-GC referrals<br>-GT undertaken<br><br>*Client:*<br><br>Cancer prevention<br>**-**Identification of hereditary Cancer<br><br>**CFIR**<br>*Inner setting*<br>Readiness for implementation<br>-available resources<br><br>*Process*<br>Engaging<br>– key stakeholders	
Bednar et al[39]<br><br>2019<br><br>USA	*Content:*<br><br>I: Step wise process change to increase access to GT and GC using IGC, electronic health record (EHR), education, GC apt scheduling and team meeting<br>*IGC started on 6/30/2015;*<br>• Integrate GC in Gynaecology Oncology clinic<br>• Optimize GC appointment scheduling<br>(N=9 EC).<br>*Physician education started on 12/1/2015*<br>• Physicians attend national meetings and conferences discussing hereditary cancer.<br>• GC provide education as needed.<br>Clinic patient tracking started on 1/1/2016	*Healthcare Professionals:*<br><br>Genetic counsellor<br>Gynaecologic oncologists<br>Nurses<br>Advanced practice registered nurses (APRN)	1. Recommendations for tumour testing<br>149/184 (81%)<br><br>2. Completion of tumour testing<br>I: 93/149 (62.4%)<br>C: NR (p < 0.001)<br><br>13/93 (14) having abnormal results<br><br>3.GC referral<br>I: 15/93 (16.1%)<br>C: NR	*Service:*<br>Effectiveness<br>- GT undertaken<br>- GC referral<br>- GC apt uptake<br>- TT undertaken<br><br>Equity<br>- GT access<br>- GC referrals<br>-GT/TT undertaken<br><br>*Client:*	Poor quality<br>Case series with no comparator for control

Reference	Implementation strategies and framework	Participants and Health Setting	Intervention Influence	Framework mapping	Quality and method
	• Research data coordinator collected data from clinic schedules and the medical record to determine whether patients received GC/GT.<br>(n=96 EC)<br>AGCR started on 1/1/2016<br>• Electronic referral to GC drafted for patients who have not had GC/GT (N=110)<br>Provider email notifications *started on 1/1/2016*<br>• Research data coordinator and GC notify physician/care team of upcoming patients not previously referred for GC/GT.<br><br>(N = 184 EC)<br><br>C: Usual care for EC tumour testing and referral to GC for EC (N = NR)<br><br>*Duration:*<br>I: June 2015 - August2017<br>C: Prior to 30.06.15<br><br>*Implementation framework:*<br>Model for Improvement quality improvement framework includes Plan-Do Study-Act (PDSA) cycles	*Healthcare Institution:*<br><br>Regional hospital – single site with a gynaecologic oncology clinic	4. GC and GT completion<br>I:12/15 (80%) completed GC<br>C: NR<br>8/12 (66%) completed GT<br>C: NR<br>No stats<br><br>5. Patients identified with mutations<br>I: 3/8 (37.5%)<br>C: NR<br>No stats	Cancer prevention<br>- Identification of hereditary Cancer<br><br>**CFIR**<br>*Inner setting*<br>Readiness for implementation<br>- access to knowledge & information<br>-available resources<br><br>*Process*<br>Engaging<br>– key stakeholders<br><br>Executing	
I = Intervention, C=Comparator, NR= Not recorded, GT = Genetic testing, TT = Tumour testing, GC = Genetic Counselling EC= endometrial cancer UTS = universal tumour screening MSI = microsatellite instability testing IHC = immunohistochemistry TNBC= triple negative breast cancer, BrCa = breast cancer, CRC= colorectal cancer, VUS= variant of unknown significance, EOC=epithelial ovarian cancer, EMR= electronic medical record, EHR= electronic health record, PARPi= poly (ADP-ribose) polymerase inhibitor					
