# Supplementary material for: Health system interventions to integrate genetic testing in routine oncology services: A systematic review
Source: PLoS One. 2021 May 19;16(5):e0250379. doi: 10.1371/journal.pone.0250379 (PMC8133413; doi:10.1371/journal.pone.0250379)
Supplement: S5 Table — (PDF) [file pone.0250379.s005.pdf]

S5 Table. Assessment of risk of bias of included cohort studies

Domain - Potential source of bias	Heald 2013[44]	Petzel 2014[46]	Cohen 2016[43]	Cohen 2016[47]	Senter 2017[34]	Hanley 2018[45]	Swanson 2018[37]	Uyar 2018[36]	Long 2018[42]	Lobo 2018[55]	Richardson 2019[54]
Representativeness of the post intervention health system or health professionals	High	High	Low	High	High	Low	High	High	Low	Low	Low
Selection of the pre intervention health system or health professionals	Low	Low	Low	Low	Low	Low	High	Low	Low	High	Low
Demonstration that outcome of interest was not present at start of study	Low	Low	Low	Low	Low	Low	Low	Low	Low	Low	Low
Study considers changes other than the intervention that could impact results	High	Low	High	High	High	High	Low	High	High	High	Low
Ascertainment of outcome	High	High	High	High	Moderate	High	High	High	High	High	High
Was follow up of patients long enough for outcomes to occur	Low	Low	Low	Moderate	Low	Low	Low	Low	Moderate	Low	Low
Same method of measurement used pre and post intervention	Low	Low	Low	Moderate	Low	Low	Low	Low	Moderate	Low	Low
Representativeness of the post intervention patients	Low	Low	Moderate	Moderate	Low	High	Low	Low	Moderate	Low	High
Selection of the pre intervention patients	Low	Low	Moderate	Moderate	Moderate	High	Low	Low	Moderate	Low	High
Patients pre and post intervention are comparable or analyses adjusted for differences	High	Low	High	High	High	High	Low	High	High	Low	Low
Analyses considered confounding due to time	High	High	High	High	High	High	High	High	High	High	High
Adequacy of follow up of patients	High	Low	High	High	Moderate	Moderate	High	Low	Low	High	High
**Overall Risk of bias**	**High**	**High**	**High**	**High**	**High**	**High**	**High**	**High**	**High**	**High**	**High**
