# Supplementary material for: Health system interventions to integrate genetic testing in routine oncology services: A systematic review
Source: PLoS One. 2021 May 19;16(5):e0250379. doi: 10.1371/journal.pone.0250379 (PMC8133413; doi:10.1371/journal.pone.0250379)
Supplement: S6 Table — (PDF) [file pone.0250379.s006.pdf]

S6 Table. Assessment of risk of bias of included case series

Domain - Potential source of bias	Plascokinska 2016[31]	George 2016[29]	Percival 2016[30]	Rahman 2017[32]	Bednar 2017[35]	Brown 2018[38]	Miesfeldt 2018[41]	Kemp 2019[40]	Bednar 2019[39]	Tutty 2019[48]	Grinedal 2020[53]	McLeavy 2020[51]	Rumford 2020[52]	Kentwell 2017[33]
Representative of all health system workers or patients impacted by intervention	Low	High	High	High	High	Low	High	High	High	High	Low	High	High	High
Ascertainment of outcome	Moderate	High	High	High	High	Moderate	High	High	High	High	High	High	High	High
Anonymously completed questionnaire /survey	NA	Moderate	Moderate	NA	NA	NA	NA	Moderate	NA	High	NA	High	NA	High
Was follow up of patients long enough for outcomes to occur	Low	Low	Low	Moderate	Low	Low	Low	Low	Low	Low	Low	Low	Low	Low
Adequacy of follow up of patients	High	Low	Low	High	Low	High	Low	High	Low	High	High	High	Low	Low
**Overall Risk of bias**	**High**	**High**	**High**	**High**	**High**	**High**	**High**	**High**	**High**	**High**	**High**	**High**	**High**	**High**

Key to overall rating: High risk of bias – high risk of bias in any domain, Moderate risk of bias – moderate or low risk of bias in all domains, no high risk domains, Low risk of bias – all domains low risk of bias, no moderate or high risk domains, NA = not applicable
