# Supplementary material for: Health system interventions to integrate genetic testing in routine oncology services: A systematic review
Source: PLoS One. 2021 May 19;16(5):e0250379. doi: 10.1371/journal.pone.0250379 (PMC8133413; doi:10.1371/journal.pone.0250379)
Supplement: S1 File — (PDF) [file pone.0250379.s007.pdf]

S1 File. References: Included studies

29. George A, Riddell D, Seal S, Talukdar S, Mahamdallie S, Ruark E,et al. Implementing rapid, robust, costeffective, patient-centred, routine genetic testing in ovarian cancer patients. Sci. Rep. 2016; doi: 10.1038/srep29506.

30. Percival N, George A, Gyertson J, Hamill M, Fernandes A, Davies E, et al. The integration of BRCA testing into oncology clinics. Br J Nurs. 2016;12:690-694

31. Plaskocinska I, Shipman H, Drummond J, Thompson E, Buchanan V, Newcombe B, et al. New paradigms for BRCA1/BRCA2 testing in women with ovarian cancer: results of the Genetic Testing in Epithelial Ovarian Cancer (GTEOC) study. J Med Genet. 2016; doi:10.1136/jmedgenet-2016-10390.

32. Rahman B, Lanceley A, Kristeleit RS, Ledermann, JA, McCormack M,et al. Mainstreamed genetic testing for women with ovarian cancer: first-year experience. J Med Genet. 2019;56:195–198.

33. Kentwell M, Dow E, Antill Y, Wrede CD, McNally O, Higgs E, et al. Mainstreaming cancer genetics: A model integrating germline BRCA testing into routine ovarian cancer clinics. Gynecol Oncol. 2017;145:130-136.

34. Senter L, O'Malley DM, Backes FJ, Copeland LJ, Fowler JM, Salani, R, et al. Genetic consultation embedded in a gynecologic oncology clinic improves compliance with guideline-based care. Gynecol Oncol. 2017;147:110–114.

35. Bednar EM, Oakley HD, Sun CC, Burke CC, Munsell MF, Westin SN, et al. A universal genetic testing initiative for patients with high-grade, non-mucinous epithelial ovarian cancer and the implications for cancer treatment. Gynecol Oncol. 2017;146 399–404.

36. Uyar D, Neary J, Monroe A, Nugent M, Simpson P, Geurts JL. Implementing a quality improvement project for universal genetic testing in women with ovarian cancer. Gynecol Oncol. 2018 doi 10.1016/j.ygyno.2018.03.0590090-8258/

37. Swanson CL, Kumar A, Maharaj JM, Kemppainen JL, Thomas BC, Weinhold MR, et al. Increasing genetic counseling referral rates through bundled interventions after ovarian cancer diagnosis. Gynecol Oncol. 2018;149:121–126.

38. Brown J, Athens A, Tait DL, Crane EK, Higgins RVR, Naumann W, et al. A Comprehensive Program Enabling Effective Delivery of Regional Genetic Counseling. Int J Gynecol Cancer. 2018;28: 996-1002.

39. Bednar EM, Sun CC, Camacho B, Terrell J, Rieber AG, Ramondetta L, et al. Disseminating universal genetic testing to a diverse, indigent patient population at a county hospital gynecologic oncology clinic. Gynecol Oncol. 2019;152: 328–333.

40. Kemp Z, Turnbull A, Yost S, Seal S, Mahamdallie S, Poyastro-Pearson E,et al. Evaluation of Cancer-Based Criteria for Use in Mainstream BRCA1 and BRCA2 Genetic Testing in Patients With Breast Cancer. JAMA Network Open. 2019; doi: 10.1001/jamanetworkopen.2019.4428

41. Miesfeldt S, Feero WG, Lucas FL, Rasmussen K. Association of patient navigation with care coordination in a Lynch syndrome screening program. Transl Behav Med. 2018 23;8:450-455.

42. Long JC, Debono D, Williams R, Salisbury E, O’Neill S, Eykman E,et al. Using behaviour change and implementation science to address low referral rates in oncology. BMC Health Serv Res. 2018 18:904

43. Cohen PA, Nichols CB, Schofield L, Van Der Werf S, Pachter N. Impact of Clinical Genetics Attendance at a Gynecologic Oncology Tumor Board on Referrals for Genetic Counseling and BRCA Mutation Testing. Int J of Gynecol Cancer. 2016;26:892-897.

44. Heald B, Plesec T, Liu X, Pai R, Patil D, Moline J,et al. Implementation of Universal Microsatellite Instability and Immunohistochemistry Screening for Diagnosing Lynch Syndrome in a Large Academic Medical Center. J Clin Oncol. 2013;31:1336-1340.

45. Hanley GE, McAlpine JN, Miller D, Huntsman D, Schrader KA, Gilks CB,et al. A population-based analysis of germline BRCA1 and BRCA2 testing among ovarian cancer patients in an era of histotype-specific approaches to ovarian cancer prevention. BMC Cancer. 2018; 18:254.

46. Petzel SV, Vogel RI, McNiel J, Leininger, A, Argenta, PA, Geller MA. Improving Referral for Genetic Risk Assessment in Ovarian Cancer Using an Electronic Medical Record System. Int J of Gynecol Cancer. 2014;24:1003-1009.

47. Cohen SA, Laurino M, Bowen DJ, Upton MP, Pritchard C, Grady WM. Initiation of Universal Tumor Screening for Lynch Syndrome in Colorectal Cancer Patients as a Model for the Implementation of Genetic Information Into Clinical Oncology Practice. Cancer. 2016; 393-401.

48. Tutty E, Petelin L, McKinley J, Young M, Meiser B, Rasmussen VM, et al. Evaluation of telephone genetic counselling to facilitate germline BRCA1/2 testing in women with high-grade serous ovarian cancer. Eur J Hum Genet. 2019; doi.org/10.1038/s41431-019-0390-9

49. Meiser B, Gleeson M, Kasparian N, Barlow-Stewart K, Ryan M, Watts K,et al. There is no decision to make: Experiences and attitudes toward treatment-focused genetic testing among women diagnosed with ovarian cancer. Gynecol Oncol. 2012;124:153-157.

50. Shipman H, Flynn S, MacDonald-Smith CF, Brenton J, Crawford R,et al. Universal BRCA1/BRCA2 Testing for Ovarian Cancer Patients is Welcomed, but with Care: How Women and Staff Contextualize Experiences of Expanded Access. J Genet Counsel. 2017; 26:1280–1291

51. McLeavy L, Rahman B, Kristeleit R, Ledermann J, Lockley M, McCormack M, et al. Mainstreamed genetic testing in ovarian cancer: patient experience of the testing process. Int J Gynecol Cancer. 2020;30:221–226.

52. Rumford M, Lythgoe M, McNeish I, Gabra H, Tookman L, Rahman N,et al. Oncologist-led BRCA 'mainstreaming' in the ovarian cancer clinic: A study of 255 patients and its impact on their management. Sci Rep. 2020 10:3390

53. Grindedal EM, Jørgensen K, Olsson P, Gravdehaug B, Lurås H, Schlichting E,et al. Mainstreamed genetic testing of breast cancer patients in two hospitals in South Eastern Norway. Fam Cancer. 2020;19:133–142.

54. Richardson M, Jung Min H, Hong Q, Compton K, Wing Mung S, Lohn Z,et al. Oncology Clinic-Based Hereditary Cancer Genetic Testing in a Population-Based Health Care System. Cancers. 2020;12:338.

55. Lobo M, López-Tarruella S, Luque S, Lizarraga S, Flores-Sánchez C, Bueno O,et al. Evaluation of Breast Cancer Patients with Genetic Risk in a University Hospital: Before and After the Implementation of a Heredofamilial Cancer Unit. J Genet Couns. 2018; 27:854–862.
