# Supplementary material for: Health system interventions to integrate genetic testing in routine oncology services: A systematic review
Source: PLoS One. 2021 May 19;16(5):e0250379. doi: 10.1371/journal.pone.0250379 (PMC8133413; doi:10.1371/journal.pone.0250379)
Supplement: S2 File — (DOCX) [file pone.0250379.s008.docx]

**S2 File. Definitions**

Various terms from the discipline of implementation science are used throughout the review and are defined (as per the Standards for Reporting Implementation Studies (StaRI) guidance^23^) as follows:

- an intervention refers to a single unit that can bring about change in a system^23^.
- an implementation strategy is a collection of techniques that addresses a set of barriers in a system to promote the use of an evidence-based practice (EBP) or an intervention^23^.
- implementation outcomes focus on the process or quality measures to assess the impact of the implementation strategy, such as adherence to a new practice, acceptability, feasibility, adaptability, fidelity, costs and returns^21^.
- complex interventions are usually described as interventions that contain several interacting components^24^.
